# Impact of a Mobile Money–Based Conditional Cash Transfer Intervention on Health Care Utilization in Southern Madagascar: Mixed-Methods Study

**DOI:** 10.2196/60811

**Published:** 2025-03-03

**Authors:** Mara Anna Franke, Anne Neumann, Kim Nordmann, Daniela Suleymanova, Onja Gabrielle Ravololohanitra, Julius Valentin Emmrich, Samuel Knauss

**Affiliations:** 1 Charité Center for Global Health, Charité - Universitätsmedizin Berlin, Charitéplatz1, Berlin, 10117, Germany, 49 15754821334; 2 RWTH Aachen, Aachen, Germany; 3 Sciences Po, Institut d'Etudes Politiques de Paris, Paris, France; 4 Doctors for Madagascar, Antananarivo, Madagascar

**Keywords:** cash transfer intervention, Madagascar, Sub-Saharan Africa, health care utilization, humanitarian assistance, Africa, mobile, mixed methods study, money, quantitative, qualitative, thematic analysis, policy, service, delivery, health care system, cash, economic, financial, payment, time series

## Abstract

**Background:**

Mobile money–based cash transfer interventions are becoming increasingly utilized, especially in humanitarian settings. southern Madagascar faced a humanitarian emergency in 2021-2022, when the second wave of the COVID-19 pandemic and a severe famine affected the fragile region simultaneously.

**Objective:**

This mixed-methods study aims to analyze the impact and factors influencing the success of a mobile money–based conditional cash transfer intervention for health care utilization at 4 primary and 11 secondary facilities in Madagascar.

**Methods:**

We obtained quantitative data from 11 facility registers, detailing patient numbers per month, categorized into maternity care, surgical care, pediatric care, outpatient care, and inpatient care. An interrupted time series analysis, without a control group, was conducted using the end of the intervention in July 2022 as the cut off point. For qualitative data, 64 in-depth interviews were conducted with health care providers, NGO staff, policymakers, beneficiaries, and nonbeneficiaries of the intervention, and was interpreted by 4 independent researchers using reflexive thematic analysis to identify facilitators and barriers to implementation.

**Results:**

The interrupted time series analysis showed a significant negative impact on health care utilization, indicating a reduction in health care–seeking behavior after the end of the cash transfer intervention. The effect was stronger in the slope change of patient numbers per month (defined as *P*<.05), which significantly decreased in 39 of 55 (70%) models compared to the step change at the end of the intervention, which showed a significant but lower change (*P* <.05) in 40% (22/55) of models. The changes were most pronounced in surgical and pediatric care. The key factors that influenced the success of the implementation were grouped across three levels. At the community level, outreach conducted to inform potential beneficiaries about the project by community health workers and using the radio was a decisive factor for success. At participating facilities, high intrinsic staff motivation and strong digital literacy among facility staff positively influenced the intervention. Confusion regarding previous activities by the same implementing NGO and perceptions of unfair bonus payments for health care providers included in the project negatively affected the intervention. Finally, at the NGO-level, the staff present at each facility and the speed and efficiency of administrative processes during the intervention were decisive factors that influenced the intervention.

**Conclusions:**

The conditional cash transfer intervention was overarchingly successful in increasing health care utilization in southern Madagascar in a humanitarian setting. However, this success was conditional on key implementation factors at the community, facility, and NGO levels. In the future, similar interventions should proactively consider the key factors identified in this study to optimize the impact.

## Introduction

Over the past decade, Sub-Saharan Africa (SSA) has seen a substantial rise in the number of people with access to digital technologies. Digital transformation has made a stark difference for previously disenfranchised communities, especially in the field of financial services [1].

One technology that stands out is mobile money, which allows the creation of bank accounts and enables financial transactions using unstructured supplementary service data codes without requiring an internet connection [1]. The use of mobile money has seen an unprecedented rise in SSA over the past years, with over 781 million users in 2022 [2].

Mobile money and digital payment services are increasingly used in providing humanitarian and development aid, including by large multilateral organisations such as the World Bank [3]. The COVID-19 pandemic has resulted in a boost in mobile money–based support schemes, aiming to ensure equitable access to health care during crises [4]. Mobile money–based interventions are particularly feasible in humanitarian settings as they are fast to implement, cost-efficient, and reach people who are structurally excluded or in hard-to-reach areas [1,5].

However, scientific evidence on the effectiveness of such programs in increasing access to health care is scarce. Similarly, research on implementation factors that influence their effectiveness is lacking.

Madagascar, with a population of 29 million [6], experienced two humanitarian crises in 2021 and 2022. First, the COVID-19 pandemic led to a reduction in GDP, increased unemployment, and poverty [7] Second, a severe drought and subsequent famine affected the southern regions of the island, where extreme poverty and malnutrition rates are particularly high [8,9]. Health care access in Madagascar—particularly in the south was already limited before these crises, due to a severely resource-constrained health system and high financial barriers to care [10]. In 2021 and 2022, the additional economic strain from the pandemic and famine put the financial viability of health facilities at risk, which rely on patients’ out-of-pocket payments [11,12]. In response, the nongovernmental organisation (NGO), Doctors for Madagascar implemented a mobile money–based conditional cash transfer intervention to improve access to health care and financial stability of health facilities.

In this study, we aim to (1) analyze the impact of this intervention on health care utilization in a humanitarian setting, and (2) to identify factors contributing to its success.

We expect this research to provide evidence for policymakers and implementers of future health care interventions in humanitarian settings.

## Methods

### Study Setting

Madagascar is one of the least developed countries globally [13]. The country has a maternal mortality rate of 392 deaths per 100,000 live births, an under-5 mortality rate of 71 per 1000 live births, and an average life expectancy of 64 years [6]. Over 40% of health care expenses are paid out-of-pocket [14]. With over 80% of the population living in extreme poverty, financial barriers are a major hindrance to accessing health care [6,8].

This study draws on data from selected health facilities in 7 regions of Madagascar (see Multimedia Appendix 1), where extreme poverty rates range between 78% and 95% [8]. During the intervention, the region was hit by severe drought and famine, putting over 2 million people into acute food insecurity [9]. Simultaneously, the second wave of the COVID-19 pandemic affected the economy severely, leading to a 7% decrease in GDP and a nationwide recession [7,15].

### Intervention Description

This study analyzed a mobile money–based conditional cash transfer intervention, aimed at increasing health care utilization; it was implemented from February 2021 to July 2022. The inclusion criteria for patients to benefit from the intervention were (1) presenting for care at a participating facility during the intervention timeframe, (2) belonging to one of the eligible patient groups (detailed below), and (3) registering on the digital mTOMADY platform with a mobile money account ( support was available at the health facilities at point of care). The intervention covered 80% of patients’ expenses for medications and medical consumables through conditional cash transfers. Patients qualified for the intervention if they sought care for (1) acutely life-threatening conditions, (2) accidents or injuries, (3) pregnancy, childbirth, or postpartum care, or (4) pediatric care. The decision to include an eligible patient in the intervention lied with the treating health care provider, and patients could decline participation at any point.

The costs covered by the intervention were limited to medical consumables and medication due to donor requirements. It did not cover consultation fees, laboratory fees (excluding laboratory consumables), hospitalization, or indirect expenses (eg, transportation costs).

Facilities could express their interest in participating with the implementing NGO, which selected facilities based on previous collaborative experience and geographical location, prioritizing those in underserved regions with high poverty rates. The NGO staff members were employed at each participating facility to support the administration of the intervention.

Once a patient completed treatment, the health facilities submitted claims for reimbursement through a digital platform for health care–related payments developed and provided by the German-Malagasy NGO, mTOMADY [16]. The claims were filed by a local employee, usually an administrative clerk at the health facility. Claims contained patient sociodemographic data (eg, age, gender, family size), medical information (diagnoses, symptoms, quantities of medication received, and consumables used), and cost data (eg, prices of each type of medication and medical consumables). All claims underwent scrutiny for accuracy by a team of registered doctors at the implementing NGO’s central administrative level. Clarification was requested by this team in case of inconsistencies or missing data. Health facilities were reimbursed for approved claims using mobile money. Participating facilities received a small bonus payment (approximately USD 0.50) for each approved claim.

### Data Sources

#### Quantitative Data

The primary data sources for the interrupted time series (ITS) analysis were routine facility-level registers from January 2021 to December 2022. These registers were maintained by health facilities for reporting to the national health information system. The registers categorized patient numbers per month into outpatient, inpatient, surgical, maternity, and pediatric care. The register data were obtained from 11 of the 15 facilities that participated in the intervention; 4 facilities declined to share their data. One facility provided only total patient numbers per month rather than disaggregated data. The facilities compiled register data from paper-based registers into digital datasheets. We received these datasheets, which were screened for plausibility and outliers by an independent researcher. Clarification was sought from the facilities in case of inconsistencies. Cleaned data sheets from each facility were combined into one data sheet for analysis.

#### Qualitative Data

Qualitative data were collected through interviews with health care providers, project implementation staff, policymakers, beneficiaries and nonbeneficiaries (individuals who were eligible to participate in the intervention but who opted out) of the intervention . Interviews were conducted between September 9 and November 11, 2022. Tailored interview guides were developed for each participant group to capture their unique perspectives and experiences.

Health care providers were recruited through phone calls or direct visits. We sampled facilities across different regions, including a mix of primary, secondary, public, private, and faith-based providers. Project implementation staff were recruited through phone calls or emails. Policymakers were purposively sampled and contacted via telephone or email, with additional participants identified through snowball sampling. Beneficiaries and nonbeneficiaries were approached in person within their communities with the help of community health workers (CHWs). Communities were selected using a purposive sampling approach, to represent two communities each in proximity and farther from the 2 facilities where the intervention was implemented for the longest duration.

A Malagasy researcher fluent in local dialects and French, conducted the interviews after undergoing comprehensive training in qualitative research and ethics. The study’s purpose and objectives were communicated to the participants in their preferred language, and written informed consent was obtained. Interviews were conducted in private settings chosen by participants, in either Malagasy or French, based on participant preference, and were audio-recorded with consent.

### Data Analysis

We used a convergent mixed-methods design for this study, in which qualitative data were analyzed based on the findings from the quantitative analysis [17]. We analyzed qualitative data to explain the differences in the impact of the conditional cash transfer intervention on health care utilization at participating facilities.

#### Quantitative Data

We conducted an ITS analysis using segmented linear regression without a control group. To assess nonstationarity, we used the Durbin-Watson test to evaluate and adjust for autocorrelation [18]. We did not assess for seasonal trends in the data. Our dataset covered only 2 years, and this limited period and number of data points did not allow for a comprehensive analysis of seasonality. We used the month in which the intervention ended at each facility as the cutoff point and analyzed step and slope changes at that point. For all facilities, the preintervention period was defined as the time from when they joined the intervention until the end of May 2022 (except for one facility, which discontinued the intervention in September 2021). The postintervention period ranged from June 2022 to December 2022 (except for one facility, for which the postintervention period began in October 2021). The start date of the intervention was different for each facility, ranging from March 2021 to December 2021.

We developed separate models per facility for all patients and by patient subgroups (outpatient care, inpatient care, surgical care, maternity care, and pediatric care). As facilities were onboarded to the intervention on a rolling basis and one facility left the intervention early, we did not run a model encompassing all facilities. All statistical analyses were conducted using R Studio, (version 2023.06.1, R Foundation for Statistical Computing) [19]. A *P* value of <.05 was considered statistically significant.

#### Qualitative Data

For qualitative data, recordings were transcribed verbatim and translated into English by trained interpreters. To ensure accuracy, a native Malagasy speaker conducted random spot checks, comparing transcripts and translations to the original recordings. Researchers anonymized all identifying information before transcription.

Data were securely stored in a password-protected digital database. Four researchers independently coded all interviews using reflexive thematic analysis [20]. Regular meetings were held to ensure coding validity and consistency. We used NVivo (version 12, Lumivero) for all qualitative analyses [21].

### Ethical Considerations

Ethical approval for all components of the study was obtained from the University of Heidelberg Ethics Committee (Heidelberg, Germany) under the registration number: S-982/2021. In addition, we obtained formal approval from the district health office, a regional subdivision of the Malagasy Ministry of Health, in each district in which data were collected. For all secondary analyses of patient data, the ethics committee waived additional informed consent as all data were anonymized before being forwarded to the research team. For primary data collection for qualitative interviews, written informed consent was obtained from each participant prior to the interview. All identifying data were removed from the interviews during transcription, and all interviews were pseudonymized before analysis. Participants received no compensation for their participation in this study.

We referred to the STROBE (Strengthening the reporting of observational studies in epidemiology) and SRQR (Standards for reporting qualitative research) reporting guidelines in the preparation of this manuscript [22,23].

## Results

### Sample Description

We obtained register data from 11 out of 15 health facilities; 4 facilities refused to share their register data. Multimedia Appendix 2 contains details on these 11 facilities.

For the qualitative data, we analyzed data from 64 interviews, which lasted between 30 minutes and 1.5 hours, with an average interview duration of 47 minutes. Ten interviews were conducted with project implementation staff, 22 with health care providers, 17 with beneficiaries, 9 with nonbeneficiaries, and 6 with policymakers.

### Effect of the Intervention on Health Care Utilization

**Figure 1.**
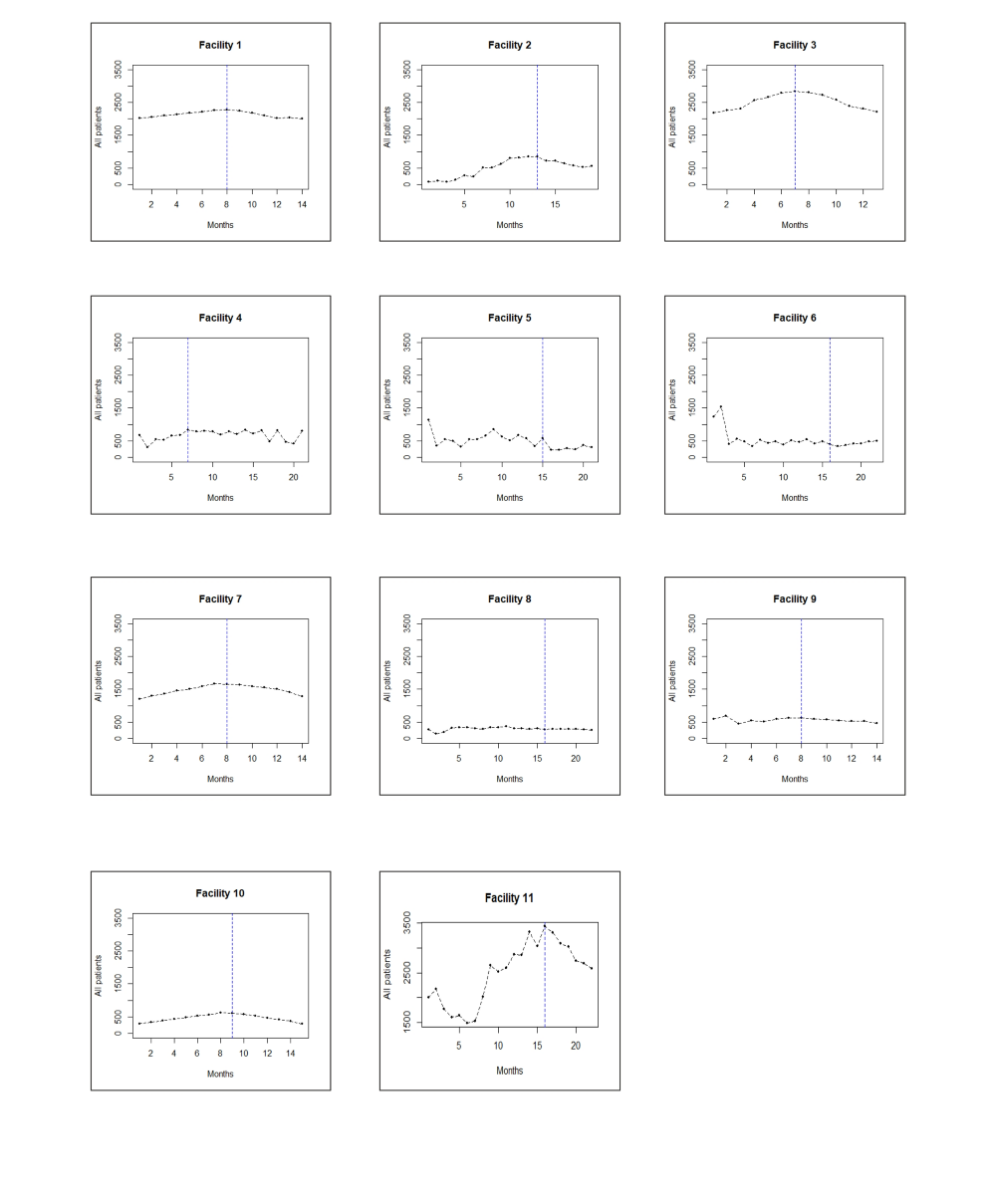
Total patient numbers for all patient categories (inpatient, outpatient, surgical, maternity, and pediatric care, depending on services offered by each facility) at 11 health facilities in southern Madagascar that participated in a mobile money–based conditional cash transfer intervention for health care utilization between March 2021 and July 2022. The blue dotted lines mark the end of the intervention at each facility.

Overall, health care utilization decreased significantly across most facilities after the end of the intervention. The end of the intervention had a more pronounced effect on patient numbers per month in the long term, which decreased significantly in 39 of 55 (70%) ITS models across separate facilities and patient groups. In comparison, the point effect in patient numbers only showed a significant decrease in 40% (22/55) of all models. Figure 1 illustrates total patient numbers per month at each facility.

The facility with the most pronounced decrease in patient numbers after the end of the intervention was Facility 10, a private facility located in an urban area. Overall, the negative effects of the end of the intervention were most pronounced in maternity, pediatric, and surgical care; whereas, outpatient consultations were the least affected by the end of the intervention.

Table 1 shows the detailed results of the ITS models. The step change values indicate the immediate difference in patient numbers at the end of the intervention, whereas the slope change describes the change in patient numbers per month over time.

**Table 1.** Results of interrupted time series analysis using interrupted linear regression, evaluating changes in health care utilization measured by patient numbers per month across 11 health facilities in southern Madagascar after the end of a mobile money-based conditional cash transfer intervention..

Facilitycategories	Step change in patient numbers per month at end of intervention	*P* value^a^	Slope change in patient numbers per month after end of intervention	*P* value^a^
Facility 1				
All patients	−2.84	.02	−13.134	<.001
Outpatient consultations	−0.61	.56	−3.77	.002
Internal medicine	−2.75	.02	−13.59	.008
Surgical care	−9.10	.007	−53.94	<.001
Maternity care	−3.08	.01	−16.75	<.001
Pediatric care	−2.86	.02	−14.60	.002
Facility 2				
All patients	0.22	.83	−9.43 )	<.001
Outpatient consultations	−4.83	0.004	−14.09	<.001
Internal medicine	0.30	.77	−7.51	.002
Maternity care	−3.78	<.001	−14.38	<.001
Pediatric care	1.34	.20	−3.71	<.001
Facility 3				
All patients	−4.12	0.001	−15.95	.007
Outpatient consultations	−2.36	.04	−9.17	<.001
Internal medicine	−4.69	0.003	−7.26	.003
Surgical care	−3.66	<.001	−21.22	<.001
Maternity care	−0.18	.86	−15.28	<.001
Pediatric care	−1.82	.10	−7.17	<.001
Facility 4				
All patients	−1.77	.09	−1.35	.19
Outpatient consultations	−1.77	.09	−1.35	.19
Internal medicine	−1.35	.19	−1.14	.27
Pediatric care	−2.69	.02	−1.47	.16
Facility 5				
All patients	0.69	.49	−0.27	.79
Outpatient consultations	0.71	.49	−0.24	.81
Maternity care	−0.65	.53	−1.79	.09
Facility 6				
All patients	−0.01	.99	1.24	.23
Facility 7				
All patients	−2.12	.06	−11.88	<.001
Outpatient consultations	−1.34	.21	−4.99	<.001
Internal medicine	−3.33	.003	−14.55	<.001
Surgical care	2.42	.04	−6.74	<.001
Maternity care	−0.70	.49	−8.51	<.001
Pediatric care	−2.49	.03	−20.23	<.001
Facility 8				
All patients	1.01	.33	−0.79	.43
Outpatient consultations	0.82	.42	−0.77	.45
Internal medicine	0.21	.83	1.97	.06
Surgical care	−0.39	.69	−0.33	.75
Maternity care	0.99	.33	0.93	.37
Pediatric care	−3.65	.007	−3.07	.005
Facility 9				
All patients	−1.12	.29	−1.38	.19
Outpatient consultations	−0.41	.69	0.19	.86
Internal medicine	−0.79	.45	−3.08	.01
Surgical care	−1.72	.12	−1.64	.13
Maternity care	−1.71	.12	2.06	.07
Pediatric care	−0.99	.34	0.34	.74
Facility 10				
All patients	−7.13	.002	.07−49.69	<.001
Outpatient consultations	−2.03	.07	−12.65	.004
Internal medicine	−3.67	.006	−23.29	<.001
Surgical care	−0.14	.89	−8.50	<.001
Maternity care	−3.17	<.001	−28.98	<.001
Pediatric care	−5.43	.004	−21.03	<.001
Facility 11				
All patients	−1.90	.07	−2.72	.01
Outpatient consultations	−0.07	.95	−4.74	.006
Internal medicine	−2.61	.02	−1.69	.11
Surgical care	0.91	.38	−2.14	.04
Maternity care	−0.51	.62	−3.69	.004
Pediatric care	−0.88	.39	−2.62	.02

a *P<.05 was considered statistically significant.*

### Explanatory Qualitative Findings

The qualitative interviews elucidated 7 key factors that differed between facilities where the ending of the intervention had a significant impact on health care utilization and those where it did not. These factors spanned across 3 levels: the community level, the facility level, and the level of the implementing NGO. Table 2 summarizes these factors, as determined in the qualitative analysis of in-depth interviews with key implementation stakeholders, sorted by factors and facilities, with and without significant changes in health care utilization after the end of the conditional cash transfer intervention.

**Table 2.** Key factors influencing the successful implementation of the digital cash transfer intervention..

Key factors	Facilities with significant effects^a^	Facilities without significant effects^b^
Community level		
Community Outreach	Where knowledge about the intervention was high, community members were more likely to seek care	A lack of knowledge about the intervention led to confusion and insecurity and a hesitation to utilize health care.
Facility level		
Facility staff motivation	Facilities where staff expressed high inherent motivation and altruistic mindset performed better and were more adaptive to programmatic challenges.	Facilities, where staff expressed lower intrinsic motivation, were less adaptive to programmatic challenges and performed worse.
Bonus payments paid by the NGO^c^	Bonus payments were perceived as useful to increase motivation.	Expressed that bonus payments were insufficient and demotivating, especially when unequally distributed among facility staff
Preexisting activities of the same implementing NGO	Facilities without previous interventions from the same NGO did not experience confusion about previous interventions which could thus not impact their work.	Confusion regarding the overlap and differences of the conditional cash transfer intervention with previous NGO activities led to a lack of clarity for health care providers.
Digital literacy of facility staff	High levels of digital literacy among facility staff positively impacted intervention uptake.	Low levels of digital literacy among facility staff negatively impacted intervention uptake.
NGO level		
NGO staff present at each facility	Presence of NGO staff at each facility was perceived as useful.	Challenges when NGO staff were absent, for example, on weekends or when they served multiple facilities
Speed and ease of the intervention’s administrative processes	Facilities that were onboarded later in the intervention benefited from decreased challenges as frequent issues experienced during the first few months of the intervention had been resolved.	Delays and challenges in the programmatic process of the intervention (especially claims validation and reimbursement) were major barriers, especially for the first facilities onboarded.

a Facilities F1, F2, F3, F10, F11.

b Facilities F4, F5, F6, F8, F9.

c NGO: nongovernmental organization.

### Community Level

#### Community Outreach

For beneficiaries, different perceptions of the outreach conducted as part of the intervention and their subsequent knowledge about the intervention influenced health care utilization. Outreach about the intervention was mostly conducted by CHWs, who held group discussions, mass outreach activities (eg, during market days), or conducted home visits and explained the intervention and its procedures to the population. These activities were usually combined with health education activities conducted by CHWs. Additionally, brief radio spots describing the intervention were broadcast in the intervention area. Additionally, at higher-performing facilities, patients expressed that hearing about the intervention through CHWs or on the radio encouraged them to seek care. Conversely, participants in proximity to lower-performing facilities expressed a lack of information about the intervention and often only heard about it once they had arrived at a health facility.

*It was a community health worker who talked to me and explained the intervention. (He said,) that we have to go to the hospital when we are sick and not go anywhere (else) for care.*

[Beneficiary 5]

*There is no awareness (raising) here. I did not hear (about the intervention)*.

[Nonbeneficiary 7]

### Facility Level

#### Facility Staff Motivation

The intrinsic motivation of individual health care providers was different between facilities. Facilities that actively sought to participate in the intervention and where health care providers had a strong interest in its success performed better. At these facilities, health care providers took considerable steps to ensure the functioning of the intervention, including covering patient copayments out of pocket or using personal phones to enroll patients.

For the facilities that received it involuntarily, we found several kinds of problems because like any project if there is no commitment from the people it is not possible to accomplish it. For the facilities that accepted it, these facilities welcomed it with their hearts, and we immediately saw that the project worked well and that it brought good to the whole facility.

[Project implementation staff 6]

#### Bonus Payments Paid by the NGO

With the intervention, facilities received a bonus payment for each claim. At some facilities, the bonus payment was perceived as useful to increase motivation among health care providers, while others expressed that the bonus payments were insufficient.

How bonus payments were distributed among staff members (ie, if everyone got a share or the money was kept by the individual responsible for filing the claims) further contributed to the frustration regarding the bonus payment and lower performance at some facilities.

*The intervention’s close collaborators benefited because they received indemnities, and they did their work well. I saw them running around doing their work and they did it perfectly.*

[Health care provider 3]

*At the time of the intervention, the work was voluminous; the indemnity for the agents is not sufficient. This is the problem. We work here; we work at night when there are many patients.*

[Health care provider 15]

#### Preexisting Activities of the Same Implementing NGO

Overall, facilities where the intervention was more successful in increasing health care utilization were those that had not previously collaborated with the NGO. Facilities that had previously collaborated with the NGO expressed confusion about the differences between the new intervention and interventions implemented by the same NGO previously. The data further indicated an unwillingness to adapt to the new intervention’s processes among some facility staff who had worked on these previous projects.

#### Digital Literacy of Facility Staff

The confidence and skills of health care providers and administrative clerks in using the digital tools required for the administration of the intervention equally influenced the success of the intervention. Facilities where staff had higher levels of digital literacy found the intervention easier to implement than those with lower digital literacy.

### NGO Level

#### NGO Staff Presence at Each Facility

At high-performing facilities, active NGO staff members encouraged the uptake of the intervention by patients and aided the facilities in managing the intervention-associated workload. Because of this positive role of NGO staff, their absence was perceived as difficult. Some NGO staff served several facilities, which caused issues for facilities on the days when NGO staff were absent. Emergencies that occurred at facilities during the night or on the weekends were perceived as equally challenging because the NGO staff were absent.

Further, NGO staff placed at the facilities received a salary from the NGO, whereas the facility employees who also supported the intervention did not receive an additional salary, leading to dissatisfaction among some health care providers.

*I encourage each patient who arrives there to discuss directly with the doctor and tell him that we have heard this and that. I did not also do a wait-and-see attitude, but I did everything, even if it is not my job, to win the hearts of these facilities, so that they recognise that I am enthusiastic about collaborating.*

[Project implementation staff 7]

#### Speed and Ease of the Intervention’s Administrative Processes

Facilities where the intervention had a significant impact on patient numbers tended to be those that joined later, likely benefiting from more established processes at the implementing NGO.

Issues related to the reimbursement of the facilities by the NGO significantly impacted the facilities, as delays in payout caused issues for the facilities related to paying their staff salaries or making prepayments for ordering medication and consumables. These delays were a significant source of frustration.

*The payment made by (the NGO) was a bit delayed at the beginning. It was difficult for the hospital at that time. The hospital needs to run while the cash, the money was blocked there. It was settled later on, and everything was fine.*

[Health care provider 11]

A key factor in causing the delay in the payout was the insufficient quality of the claims filed by the facilities, which was echoed by both health care providers and NGO staff. Health care providers expressed that additional training or guidelines on claim filing could have improved this situation.

Finally, early in the intervention, changes were required to the mTOMADY platform to adapt it for the intervention. These changes were perceived as confusing and challenging for facilities. However, the frequency of changes was reduced over the intervention’s lifespan, meaning that facilities onboarded later were less likely to experience such issues.

## Discussion

### Principal findings

Our study aimed to describe the impact of a mobile money–based conditional cash transfer intervention on health care utilization in a humanitarian setting, as well as to identify key factors that influenced the success of the intervention. Overall, our study showed a significant decline in health care utilization following the ending of a mobile money–based conditional cash transfer intervention in southern Madagascar. Data from 11 facilities showed a marked reduction in patient visits, particularly in maternity, pediatric, and surgical services, with outpatient consultations being the least affected. The qualitative findings highlighted key factors influencing intervention outcomes across three levels. These factors included community outreach, facility staff motivation, ease of the administrative processes of the intervention, digital literacy of facility staff, and the presence of NGO staff at facilities.

Our analysis showed that health care utilization decreased at most facilities when the conditional cash transfer intervention ended. This ties in with other findings from Madagascar, which demonstrated an increase in health care utilization when user fees were abolished [24]. This change was most pronounced for 3 patient categories: maternity patients, pediatric patients, and patients requiring surgery. Given that surgical care is particularly expensive and likely to cause catastrophic health expenditure in SSA [25-27], this is not surprising. Our findings indicate that patients might forgo necessary surgical care in the absence of financing methods for their care. The evidence on the costs of pediatric care is less comprehensive. However, several studies from SSA suggest that the costs associated with pediatric care for asthma, pediatric surgery, and pneumonia could be catastrophic for households [27-29]. Evidence on the impact of partial cost coverage or fee reduction for pediatric health services is however lacking.

Evidence on similar interventions using mobile money-based cash transfers for health care utilization in humanitarian settings is severely limited. Contrary to previous findings [30], our study revealed a significant decrease in health care utilization following the end of a cash transfer intervention. However, our study analyses a conditional cash transfer intervention, whereas previous studies examined unconditional cash transfers [30,31], in which the money may have been used for other purposes. Our findings do however align with evidence from nonhumanitarian settings, in which health care utilization increased in the presence of targeted cash transfer interventions [32,33], especially for maternity services [34]. Cash-transfer interventions in nonhumanitarian settings, especially in combination with other interventions such as health education or text-based reminders, have been shown to improve risky sexual behavior [35], and are perceived as useful in improving the adherence and management of patients with TB [36]. It must be noted that most cash transfer interventions used physical currency, limiting the comparability of our findings [30-34].

Despite an overarching impact of the conditional cash transfer intervention on health care utilization, the impact was not significant across all facilities. We identified 7 key factors that may explain these differences across facilities.

At the community level, differences in community outreach and thus the intervention-related knowledge of the population across communities was decisive. For this intervention community outreach was only conducted in certain communities. In facilities where the NGO implemented community outreach, this was done via CHWs and radio campaigns. CHWs are vital for improving health care-related knowledge, access to health care and health outcomes in the communities they serve [37,38]. As we did not interview CHWs for this sample, we cannot further elucidate the reasons behind the differences in community outreach they conducted; however, given the evidence on factors influencing CHW performance, including training, supervision, remuneration and workload [38,39], it stands to reason that similar factors were decisive in our setting.

At the facility level, facility motivation and mindset were key determining factors of the success of the intervention. Provider motivation has been described as a key factor in the implementation and adoption of new practices [38,39]. It is noteworthy that the described intervention may have impacted provider motivation and thus either positively or negatively reinforced preexisting motivation levels. For example, the intervention, leading to an increase in patient numbers at the facility, may have increased provider workload which, depending on its perceived manageability, has been identified to be a key determining factor of provider motivation [39]. Equally, the intervention required health care providers to take on additional tasks and may have negatively impacted provider motivation [39].

Bonus payments received for each claim filed, were perceived very differently across facilities. At some facilities where all staff members received benefits through bonus payments, they were perceived as motivating factors. This ties in with evidence from other contexts, which have shown that bonus payments can enhance provider motivation and potentially quality of care [40,41]. This effect is however conditional on how the distribution of bonus payments was perceived among health care providers. Bonus payments perceived as unjust or unfair decrease provider motivation [41-44 ,].

Another key factor was previous NGO interventions at the same facilities, which led to confusion at the facilities with the changes in the procedures of the new intervention. Given the sizeable percentage of public services that are delivered by NGOs in SSA [45], such negative effects of past NGO interventions on future interventions should be examined more closely.

Given the digital nature of the conditional cash transfer intervention, a lack of digital literacy at the facility level was a key factor hampering the intervention. This finding ties in with findings from other studies across SSA [46-51]. In the face of rising digitalisation of health care delivery, governments, NGOs, and multilaterals, should invest in improving the digital skills of health care providers to eliminate a key barrier to the implementation of digital health interventions in the future.

Two factors emerged as decisive at the implementing NGO level. First, NGO staff placed at each facility was perceived as an important success factor for the intervention. NGO staff alleviated health care provider workloads by supporting the intervention’s administration [52]. Despite this positive effect, the fact that NGO staff were paid directly by the NGO, may have reinforced perceived inequalities in remuneration and thus furthered pre-existing frustrations [43].

Another noteworthy finding of our study is that facilities which appeared more successful were facilities, where the intervention was implemented later, and which thus benefited from more established and well-running processes. This echoes previous findings on public health interventions, which identified “implementation infrastructure,” including robust administrative systems as a key success factor [40,41]. It is vital to consider these challenges for future, similar interventions, for example by incorporating piloting phases into implementation plans to establish robust administrative processes early on.

A key challenge for cash transfer interventions, especially in humanitarian settings, is sustaining the increases in health care utilization after financial support concludes. Based on the findings from our study we propose several strategies to enhance sustainability in similar interventions.

First, strengthening local health financing systems is essential for sustaining service utilization. Future interventions could partner with local governments, private, or non-profit health insurance schemes to transition conditional cash transfers into more permanent financial assistance for vulnerable populations. Developing mechanisms for partial subsidies through national health funds or community-based health insurance schemes can support continued access to essential services without relying solely on external funding.

Second, we observed that the increased health care utilization during the intervention period partly stemmed from targeted community outreach and health education efforts. Sustained CHWs in health promotion can maintain community awareness and perceived value of health care services. Future interventions should invest in CHW training and create resources to enable communities to make informed health decisions, encouraging health care utilization even without direct financial incentives.

Finally, the data analyzed in this study contained several indications that the intervention had a positive impact on the trust expressed by patients in health care providers and the health system. This was particularly observed at facilities where health care providers helped patients in signing up for the intervention, for example by allowing them to use their ID cards to obtain a SIM card, or where they supported patients who were struggling with copayments. This improved trust is crucial as it can lead to sustained improvements in health care-seeking behavior even after financial incentives end. Future implementers should prioritise trust-building measures—such as transparent communication, continuous outreach, and patient-centred care—to cultivate a health care environment where patients feel supported and motivated to seek timely medical assistance.

### Limitations

We conducted an ITS analysis without a control group. Given the public health emergencies in southern Madagascar in recent years, which impacted health care utilization in the region, we opted against a historic control group. In 2020 the COVID-19 pandemic heavily impacted the Malagasy population and health system [7]. In 2018-2019, a severe measles epidemic [49,53] and in 2017, a plague outbreak affected the island [52,54]. Choosing control data from health facilities in a different part of the country was equally unreliable. Even though the health system is weak across Madagascar, the south of the island has particular challenges. Geographical access barriers are particularly pronounced [8], and facilities, human resources, and equipment are particularly scarce [10]. Equally, extreme poverty is more pronounced in the study regions than in the rest of the country [8,55]. Lastly, the severe drought and subsequent famine were specific to the study region and did not affect other parts of the country [9].

Four out of fifteen health facilities refused to share their facility registers with us for quantitative analysis. As facilities did not need to give a reason for refusing to share their data, we cannot eliminate the possibility that there is thus a selection bias in our final sample. However, we included facilities with varying levels of impact of the intervention on health care utilization, suggesting that the overarching results were not skewed. Our qualitative data draws on a convenience sample of facilities, which includes most but not all facilities for which a quantitative analysis was conducted, meaning we may not have captured all factors that influenced the success of the intervention.

Further, our qualitative data were collected in Malagasy and translated into English, which may have introduced translation errors. However, all data were translated by a trained interpreter with previous experience in qualitative research and spot-checked randomly for consistency between recording and translation by a native Malagasy speaker.

### Conclusions

In conclusion, our study shows that a mobile money–based conditional cash transfer intervention led to an increase in health care utilization in a humanitarian setting, particularly in surgical and pediatric cases. This indicates that similar interventions could be useful for mitigating the effects of future humanitarian crises on health care utilization. Several factors influenced the success of the intervention. Designers and implementers of future, similar interventions should proactively mitigate these factors. Additional researchers should be invested in filling the remaining evidence gaps regarding the use of mobile money–based cash transfer interventions in humanitarian settings.

## Supplementary material

10.2196/60811 Multimedia Appendix 1 Location of health facilities in southern Madagascar that participated in a mobile money–based conditional cash transfer intervention for health care utilization between March 2021 and July 2022. Facilities located in urban areas offering secondary care are marked with blue dots, facilities located in rural areas offering secondary care are marked with red triangles, and facilities located in urban areas offering primary care are marked with green squares. The capital city of Antananarivo is marked in black. Several facilities (F4, F5, F10) were in the town of Fort-Dauphin in south-eastern Madagascar, leading to a clustering of symbols in the area.

10.2196/60811 Multimedia Appendix 2 Level of care, setting, ownership, and intervention period of 11 out of 15 health facilities in southern Madagascar that participated in a mobile money-based conditional cash transfer intervention for health care utilization between March 2021 and July 2022. The remaining 4 facilities refused to share data for analysis and were excluded from this study.

10.2196/60811 Multimedia Appendix 3 French translation of the manuscript.

## ABBREVIATIONS

CHW: Community health worker
ITS: Interrupted time series
NGO: Nongovernmental organisation
SRQR: Standards for reporting qualitative research
SSA: Sub Saharan Africa
STROBE: Strengthening the reporting of observational studies in epidemiology
