# Supplementary figures and images for: Impact of a Mobile Money–Based Conditional Cash Transfer Intervention on Health Care Utilization in Southern Madagascar: Mixed-Methods Study
Source: JMIR Mhealth Uhealth. 2025 Mar 3;13:e60811. doi: 10.2196/60811 (PMC11892416; doi:10.2196/60811)

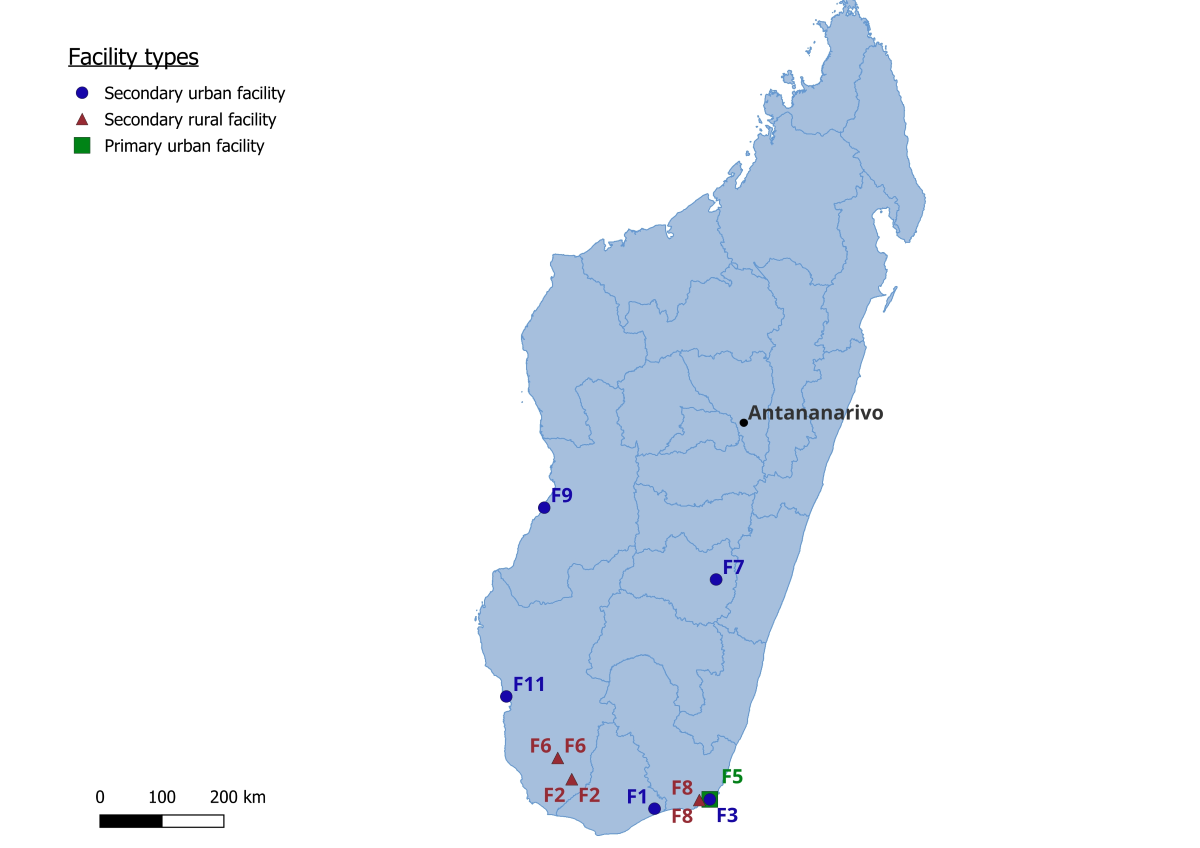

Supplement: Multimedia Appendix 1 [file mhealth-v13-e60811-s001.png]
