# Supplementary material for: Impact of a Mobile Money–Based Conditional Cash Transfer Intervention on Health Care Utilization in Southern Madagascar: Mixed-Methods Study
Source: JMIR Mhealth Uhealth. 2025 Mar 3;13:e60811. doi: 10.2196/60811 (PMC11892416; doi:10.2196/60811)
Supplement: Multimedia Appendix 2 [file mhealth-v13-e60811-s002.docx]

|  | Level of care | Setting | Ownership | Pre period | Post period |
| --- | --- | --- | --- | --- | --- |
| Facility 1 | Secondary | Urban | Public | 11/21-05/22 | 06/22 – 12/22 |
| Facility 2 | Secondary | Rural | Public | 06/21-05/22 | 06/22 – 12/22 |
| Facility 3 | Secondary | Urban | Faith-based | 12/21-05/22 | 06/22 – 12/22 |
| Facility 4 | Primary | Urban | Faith-based | 04/21-09/21 | 10/21- 12/22 |
| Facility 5 | Primary | Urban | Private | 04/21-05/22 | 06/22 – 12/22 |
| Facility 6 | Secondary | Rural | Faith-based | 03/21-05/22 | 06/22 – 12/22 |
| Facility 7 | Secondary | Urban | Faith-based | 11/21-05/22 | 06/22 – 12/22 |
| Facility 8 | Secondary | Rural | Faith-based | 03/21-05/22 | 06/22 – 12/22 |
| Facility 9 | Secondary | Urban | Faith-based | 11/21-05/22 | 06/22 – 12/22 |
| Facility 10 | Secondary | Urban | Private | 10/21-05/22 | 06/22 – 12/22 |
| Facility 11 | Secondary | Urban | Public | 03/21-05/22 | 06/22 – 12/22 |
