# Supplementary material for: Impact of a Mobile Money–Based Conditional Cash Transfer Intervention on Health Care Utilization in Southern Madagascar: Mixed-Methods Study
Source: JMIR Mhealth Uhealth. 2025 Mar 3;13:e60811. doi: 10.2196/60811 (PMC11892416; doi:10.2196/60811)
Supplement: Multimedia Appendix 3 [file mhealth-v13-e60811-s003.docx]

**Titre :** Impact d'une intervention de transfert monétaire conditionnel et digitale sur l'utilisation des soins de santé dans le sud de Madagascar - une étude de méthodes mixtes.

**Auteurs :** Mara Anna Franke1,2*, Anne Neumann1,2, Kim Nordmann3, Daniela Suleymanova4, Onja Gabrielle Ravololohanitra5, Julius Valentin Emmrich1,2,6,7†, Samuel Knauss1,6,7†

Auteur correspondant :

Mara Anna Franke, mara-anna.franke@charite.de, +49 177 6326078

**RÉSUMÉ**

**Contexte**

Les transferts monétaires basés sur le paiement mobile sont de plus en plus utilisés, en particulier dans les situations humanitaires. Le sud de Madagascar a constitué une urgence humanitaire en 2021/2022 lorsque la deuxième vague de la pandémie de COVID-19 et une grave famine ont touché simultanément cette région fragile.

**Objectif de l'étude**

Cette étude de méthodes mixtes vise à analyser l'impact et les facteurs influençant le succès d'une intervention de transfert conditionnel d'argent basée sur l'argent mobile pour l'utilisation des soins de santé dans quatre centres de santé primaires et onze établissements secondaires à Madagascar.

**Méthodes utilisées**

Nous nous sommes appuyés sur des données quantitatives provenant de onze registres d'établissements, détaillant le nombre de patients par mois, répartis par catégories (soins de maternité, soins chirurgicaux, soins pédiatriques, soins ambulatoires et soins hospitaliers). Nous avons effectué une analyse de séries temporelles interrompues, sans groupe de contrôle, en utilisant la fin de l'intervention en juillet 2022 comme point limite. Pour les données qualitatives, nous nous sommes appuyés sur les données de 63 entretiens approfondis menés avec des prestataires de soins de santé, du personnel de l'organisation non gouvernementale (ONG) chargée de la mise en œuvre, des décideurs politiques, des bénéficiaires et des non-bénéficiaires de l'intervention, interprétées par quatre chercheurs différents à l'aide d'une analyse thématique réflexive. Pour la composante qualitative, nous avons mené 63 entretiens approfondis avec des prestataires de soins de santé, du personnel d'ONG, des décideurs politiques, des bénéficiaires et des non-bénéficiaires, analysés par quatre chercheurs indépendants au moyen d'une analyse thématique réflexive afin d'identifier les facilitateurs et les obstacles à la mise en œuvre de l'intervention.

**Résultats**

L'analyse des séries temporelles interrompues a montré un impact négatif significatif de la fin de l'intervention sur l'utilisation des soins de santé, indiquant une réduction de la recherche de soins de santé après la fin de l'intervention. L'effet était plus marqué sur le changement de pente du nombre de patients par mois, qui a diminué de manière significative dans 39 des 55 (70 %) modèles (valeur P < 0,05), par rapport au changement d'échelon à la fin de l'intervention, qui n'a montré un changement significatif (valeur P < 0,05) que dans 40 % (22/55) des modèles. Les changements étaient plus prononcés pour les soins chirurgicaux et pédiatriques.

Les facteurs clés qui ont influencé le succès de la mise en œuvre ont été regroupés sur trois niveaux. Au niveau communautaire, la sensibilisation des bénéficiaires potentiels au projet par les agents de santé communautaires et la radio a été un facteur de réussite décisif. Dans les établissements participants, la forte motivation intrinsèque du personnel et son niveau élevé de connaissances numériques ont eu une influence positive sur l'intervention. La confusion concernant les activités antérieures de la même ONG chargée de la mise en œuvre et le sentiment que les primes versées aux prestataires de soins de santé inclus dans le projet étaient injustes ont eu une incidence négative sur l'intervention. Enfin, du côté de l'ONG chargée de la mise en œuvre, le personnel de l'ONG présent dans chaque établissement et la rapidité et la facilité des processus administratifs de l'intervention sont apparus comme des facteurs décisifs qui ont influencé l'intervention.

**Conclusion**

L'intervention de transfert monétaire conditionnel a globalement réussi à augmenter l'utilisation des soins de santé dans le sud de Madagascar dans un contexte humanitaire. Cependant, ce succès était conditionné par des facteurs clés de mise en œuvre au niveau de la communauté, de l'établissement et de l'ONG. Les futures interventions similaires devraient prendre en compte de manière proactive les facteurs clés identifiés.

**Mots clés**

Intervention de transfert monétaire, Madagascar, Afrique subsaharienne, utilisation des soins de santé, aide humanitaire

**INTRODUCTION**

Au cours de la dernière décennie, l'Afrique subsaharienne (ASS) a connu une augmentation substantielle du nombre de personnes ayant accès aux technologies numériques. Dans le domaine des services financiers en particulier, la transformation numérique a fait une grande différence pour des communautés auparavant privées de leurs droits [1].

L'une des technologies les plus remarquables est l'argent mobile, qui permet de créer des comptes bancaires et d'effectuer des transactions financières à l'aide de codes USSD, sans avoir besoin d'une connexion internet [1]. L'argent mobile a connu un essor sans précédent en Afrique subsaharienne au cours des dernières années : plus de 781 millions de personnes utilisaient cette technologie en 2022 [2].

L'argent mobile et les services de paiement numérique sont de plus en plus utilisés dans le cadre de l'aide humanitaire et de l'aide au développement, y compris par de grandes organisations multilatérales telles que la Banque Mondiale [3]. La pandémie de COVID-19 a entraîné une augmentation des programmes de soutien basés sur l'argent mobile, visant à garantir un accès équitable aux soins de santé en temps de crise [4]. Les interventions basées sur l'argent mobile sont particulièrement adaptées aux situations humanitaires car elles sont rapides à mettre en œuvre, rentables et permettent d'atteindre des personnes structurellement exclues ou situées dans des zones difficiles d'accès [1,5].

Cependant, les preuves scientifiques de l'efficacité de ces programmes pour améliorer l'accès aux soins de santé sont rares. De même, la recherche sur les aspects de la mise en œuvre qui influencent l'efficacité fait défaut.

Madagascar, avec une population de 29 millions d'habitants [6], a subi deux crises humanitaires en 2021 et 2022. D'une part, la pandémie de COVID-19 a entraîné une réduction du produit intérieur brut (PIB) et une augmentation du chômage et de la pauvreté [7]. D'autre part, une grave sécheresse et la famine qui s'en est suivie ont touché les régions méridionales de l'île, où les taux d'extrême pauvreté et de malnutrition sont particulièrement élevés [8,9]. L'accès aux soins de santé à Madagascar, en particulier dans le sud de l'île, était déjà limité auparavant, car le système de santé est extrêmement limité en ressources et les obstacles financiers aux soins sont élevés [10]. En 2021 et 2022, les pressions économiques supplémentaires liées à la pandémie et à la famine ont mis en péril la viabilité financière des établissements de santé, qui dépendent des paiements directs des patients [11,12]. Dans ce contexte, l'organisation non gouvernementale (ONG) Doctors for Madagascar a mis en œuvre une intervention de transfert monétaire conditionnel basé sur l'argent mobile afin d'améliorer l'accès aux soins de santé et la stabilité financière des établissements de santé.

Dans cette étude, nous visons à i) analyser l'impact de cette intervention sur l'utilisation des soins de santé dans un contexte humanitaire, et ii) identifier les facteurs contribuant au succès de l'intervention.

Nous espérons que cette recherche fournira des éléments probants aux décideurs politiques et aux responsables de la mise en œuvre de futures interventions en matière de soins de santé dans des contextes humanitaires.

**MÉTHODES**

**Cadre de l'étude**

Madagascar est l'un des pays les moins développés au monde [13]. Le taux de mortalité maternelle y est de 392 décès pour 100 000 naissances vivantes, le taux de mortalité des enfants de moins de 5 ans de 71 pour 1 000 naissances vivantes et l'espérance de vie moyenne est de 64 ans [6]. Plus de 40 % des dépenses de santé sont payées de leur poche [14]. Avec plus de 80 % de la population vivant dans l'extrême pauvreté, les barrières financières constituent un obstacle majeur à l'accès aux soins de santé [6, 8].

Cette étude s'appuie sur des données provenant d'établissements de santé sélectionnés dans sept régions de Madagascar (voir annexe 1), où les taux d'extrême pauvreté varient entre 78 % et 95 % [8]. Au cours de l'intervention, la région a été frappée par une grave sécheresse et une famine qui ont plongé plus de deux millions de personnes dans une situation d'insécurité alimentaire aiguë [9]. Simultanément, la deuxième vague de la pandémie de COVID-19 a gravement affecté l'économie, entraînant une baisse de 7 % du PIB et une récession à l'échelle nationale [7, 15].

**Description de l'intervention**

L'intervention analysée ici était une intervention de transfert monétaire conditionnel basé sur l'argent mobile qui visait à augmenter l'utilisation des soins de santé et qui a été mise en œuvre de février 2021 à juillet 2022. Les conditions pour que les patients puissent bénéficier de l'intervention étaient les suivantes : i) se présenter pour la recherche de soins de santé dans un établissement participant pendant la période d'intervention, ii) appartenir à l'un des groupes de patients éligibles détaillés ci-après, et iii) être en mesure de s'inscrire sur la plateforme numérique mTOMADY avec un compte d'argent mobile (ce qui pouvait également être fait avec le soutien des établissements de santé au point de soins). L'intervention couvrait 80 % des dépenses des patients pour les médicaments et les consommables médicaux sous la forme d'un transfert monétaire conditionnel. Les patients étaient éligibles à l'intervention s'ils cherchaient à obtenir des soins pour i) des pathologies mettant en jeu le pronostic vital, ii) des accidents ou des blessures, iii) une grossesse, un accouchement ou des soins post-partum, ou iv) des soins pédiatriques. La décision d'inclure un patient éligible dans l'intervention revenait au prestataire de soins de santé traitant, et les patients pouvaient refuser de participer à tout moment.

Les coûts couverts par l'intervention étaient limités aux consommables médicaux et aux médicaments en raison des exigences des bailleurs. L'intervention ne couvrait pas les frais de consultation, les frais de laboratoire (à l'exclusion des consommables de laboratoire), l'hospitalisation ou les dépenses indirectes (par exemple, les frais de transport).

Les établissements pouvaient exprimer leur intérêt pour une participation avec l'ONG chargée de la mise en œuvre, qui sélectionnait les établissements sur la base d'une expérience de collaboration antérieure et de la situation géographique, en donnant la priorité aux établissements situés dans des régions mal desservies présentant des taux de pauvreté élevés. Des membres du personnel de l'ONG ont été employés dans chaque établissement pour soutenir l'administration de l'intervention.

L'annexe 1 présente l'emplacement des établissements participant à l'étude.

Une fois le traitement terminé, l'établissement de santé soumettait une demande de remboursement par le biais d'une plateforme numérique pour les paiements liés aux soins de santé développée et fournie par l'ONG germano-malgache mTOMADY [16]. Les demandes ont été déposées par un employé local de l'établissement de santé, généralement un employé administratif. Les demandes contenaient des données sociodémographiques sur les patients (par exemple, l'âge, le sexe, la taille de la famille), des informations médicales (diagnostics, symptômes, quantités de médicaments reçus et consommables utilisés) et des données sur les coûts (par exemple, les prix de chaque type de médicament et de consommables médicaux). Toutes les demandes ont été examinées par une équipe de médecins agréés au niveau administratif central de l'ONG chargée de la mise en œuvre. Cette équipe a demandé des éclaircissements en cas d'incohérences ou de données manquantes. Les établissements de santé ont été remboursés pour les demandes approuvées au moyen d'argent mobile. Les établissements participants ont reçu une petite prime (environ 0,5 dollar américain) pour chaque demande approuvée.

**Sources de données**

Données quantitatives

La principale source de données pour l'analyse des séries temporelles interrompues a été les registres de routine au niveau des établissements de janvier 2021 à décembre 2022, que tous les établissements de santé conservent pour les communiquer au système national d'information sur la santé. Ces registres détaillent le nombre de patients par mois selon les catégories suivantes : soins ambulatoires, soins hospitaliers, soins chirurgicaux, soins de maternité et soins pédiatriques. Les données des registres ont été obtenues auprès de onze des quinze établissements qui ont participé à l'intervention ; quatre établissements ont refusé de partager leurs données. Un établissement n'a pas partagé les données désagrégées mais seulement le nombre total de patients par mois. Les établissements ont compilé les données des registres papier sur une feuille de données numérique. Nous avons reçu ces mêmes feuilles de données, qui ont été examinées par un chercheur indépendant pour vérifier la plausibilité et les valeurs aberrantes. En cas d'incohérence, des clarifications ont été demandées aux établissements. Les feuilles de données nettoyées de chaque établissement ont été combinées en une seule feuille de données pour l'analyse.

Données qualitatives

Les données qualitatives ont été collectées par le biais d'entretiens avec des prestataires de soins de santé, le personnel chargé de la mise en œuvre du projet, des décideurs politiques, des bénéficiaires de l'intervention et des non-bénéficiaires de l'intervention (individus qui étaient éligibles pour participer à l'intervention mais qui ont choisi de ne pas le faire). Les entretiens ont eu lieu entre le 9 septembre et le 11 novembre 2022. Des guides d'entretien distincts ont été élaborés pour chaque groupe de participants afin de saisir leurs perspectives et expériences uniques.

Les prestataires de soins de santé ont été recrutés par le biais d'appels téléphoniques ou de visites directes. Nous avons échantillonné des établissements de différentes régions et un mélange de prestataires primaires, secondaires, publics, privés et confessionnels.

Le personnel chargé de la mise en œuvre des projets a été recruté par téléphone ou par courrier électronique. Les décideurs politiques ont été sélectionnés à dessein et contactés par téléphone ou par courrier électronique, et d'autres participants ont été identifiés grâce à un échantillonnage en boule de neige. Les bénéficiaires et les non-bénéficiaires ont été approchés en personne dans leurs communautés avec l'aide d'agents de santé communautaires (ASC). Les communautés ont été sélectionnées en tant qu'échantillon raisonné, représentant deux communautés proches et deux communautés plus éloignées de deux établissements où l'intervention a été mise en œuvre le plus longtemps.

Un chercheur malgache, parlant couramment les dialectes locaux et le français, a mené les entretiens après avoir suivi une formation complète sur la recherche qualitative et l'éthique. Le but et les objectifs de l'étude ont été communiqués aux participants dans la langue de leur choix, et un consentement éclairé écrit a été obtenu. Les entretiens se sont déroulés dans des lieux privés choisis par les participants, en malgache ou en français selon la préférence des participants, et ont été enregistrés avec le consentement des participants.

**Analyse des données**

Nous avons utilisé une méthode mixte convergente pour cette étude, où les données qualitatives ont été analysées sur la base des résultats de l'analyse quantitative [17]. Nous avons analysé les données qualitatives pour expliquer les différences d'impact de l'intervention de transfert monétaire conditionnel sur l'utilisation des soins de santé dans les établissements participants.

Données quantitatives

Nous avons réalisé une série temporelle interrompue (ITS) à l'aide d'une régression linéaire segmentée sans groupe de contrôle. Pour évaluer la non-stationnarité, nous avons utilisé le test de Durbin-Watson pour évaluer et corriger l'autocorrélation [18]. Nous n'avons pas évalué les tendances saisonnières des données. Notre ensemble de données ne couvrait que deux ans, et cette période limitée ainsi que le nombre de points de données ne permettaient pas une analyse complète de la saisonnalité.

Nous avons utilisé le mois au cours duquel l'intervention a pris fin dans chaque établissement comme point de référence et nous avons analysé les changements de pas et de pente à ce moment-là. Pour tous les établissements, la pré-période a été définie comme la période entre le moment où ils ont rejoint l'intervention et la fin du mois de mai 2022 (à l'exception d'un établissement qui a quitté l'intervention en septembre 2021). La post-période s'étendait de juin 2022 à décembre 2022 (à l'exception d'un établissement pour lequel la post-période avait déjà commencé en octobre 2021). La date de début de l'intervention était différente pour chaque établissement, allant de mars 2021 à décembre 2021.

Nous avons élaboré des modèles distincts par établissement pour tous les patients et par sous-groupes de patients (soins ambulatoires, soins hospitaliers, soins chirurgicaux, soins de maternité, soins pédiatriques). Comme les établissements ont été intégrés à l'intervention sur une base continue, et qu'un établissement a quitté l'intervention prématurément, nous n'avons pas exécuté de modèle englobant tous les établissements. Toutes les analyses statistiques ont été réalisées avec R Studio, version 2023.06.1 [19]. Une valeur P < 0,05 a été considérée comme statistiquement significative.

Données qualitatives

Pour les données qualitatives, les enregistrements ont été transcrits mot à mot et traduits en anglais par des interprètes qualifiés. Pour garantir l'exactitude des données, un locuteur natif malgache a effectué des contrôles aléatoires en comparant les transcriptions et les traductions aux enregistrements originaux. Les chercheurs ont anonymisé toutes les informations d'identification avant la transcription.

Les données ont été stockées en toute sécurité dans une base de données numérique protégée par un mot de passe. Quatre chercheurs (A.N., M.F., O.R. et D.R.) ont indépendamment codé tous les entretiens en utilisant l'analyse thématique réflexive [20]. Des réunions régulières ont été organisées pour garantir la validité et la cohérence du codage. Nous avons utilisé la version 12 de NVivo pour toutes les analyses qualitatives [21].

**Considérations éthiques**

L'approbation éthique de toutes les composantes de l'étude a été obtenue auprès du comité d'éthique de l'université de Heidelberg (Heidelberg, Allemagne) sous le numéro d'enregistrement S-982/2021 : S-982/2021 En outre, nous avons obtenu l'approbation formelle du bureau de santé du district, une sous-division régionale du ministère malgache de la Santé, dans chaque district où les données ont été collectées. Pour toutes les analyses secondaires des données des patients, le comité d'éthique a renoncé à un consentement éclairé supplémentaire car toutes les données ont été rendues anonymes avant d'être transmises à l'équipe de recherche. Pour la collecte de données primaires dans le cadre d'entretiens qualitatifs, le consentement éclairé de chaque participant a été obtenu par écrit avant l'entretien. Toutes les données d'identification ont été supprimées des entretiens lors de la transcription et tous les entretiens ont été pseudonymisés avant d'être analysés. Les participants n'ont reçu aucune compensation pour leur participation à cette étude.

Nous nous sommes référés aux lignes directrices de STROBE et de SRQR pour la préparation de ce manuscrit [22, 23].

**RÉSULTATS**

**Description de l'échantillon**

Nous avons obtenu les données des registres de onze établissements de santé sur quinze, quatre établissements ayant refusé de partager leurs données. L'annexe 2 contient des détails sur ces onze établissements.

Pour les données qualitatives, nous nous sommes appuyés sur les données de 63 entretiens qui ont duré entre 30 minutes et 90 minutes , avec une durée moyenne de 47 minutes. Dix entretiens ont été menés avec le personnel chargé de la mise en œuvre du projet, 22 avec des prestataires de soins de santé, 16 avec des bénéficiaires, neuf avec des non-bénéficiaires et six avec des décideurs politiques.

**Effet de l'intervention sur l'utilisation des soins de santé**

Dans l'ensemble, l'utilisation des soins de santé après la fin de l'intervention a diminué de manière significative dans la plupart des établissements. La fin de l'intervention a eu un effet plus prononcé sur le nombre de patients par mois à long terme, qui a diminué de manière significative dans 39 des 55 (70 %) modèles ITS que nous avons exécutés pour des établissements et des groupes de patients distincts. En comparaison, l'effet ponctuel sur le nombre de patients n'a montré une diminution significative que dans 40 % (22/55) de tous les modèles. La figure 1 ci-dessous illustre le nombre total de patients par mois dans chaque établissement.


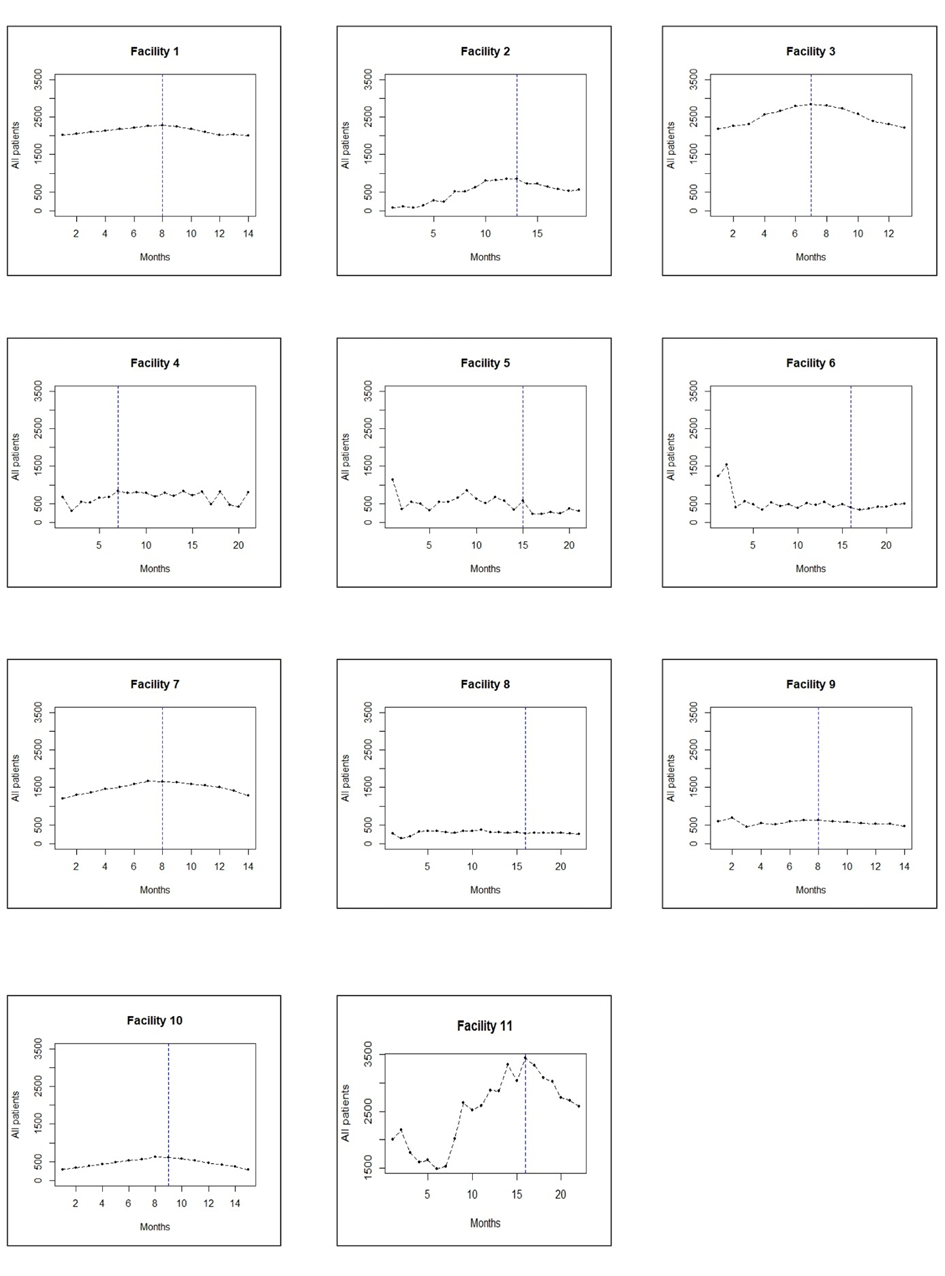


*All patients = Tous patients*

*Months = Mois*

*Facility = Centre de soins*

*Figure 1 : Nombre total de patients pour toutes les catégories de patients (soins hospitaliers, ambulatoires, chirurgicaux, maternité et pédiatriques (en fonction des services offerts par chaque établissement)) dans onze établissements de santé du sud de Madagascar qui ont participé à une intervention de transfert monétaire conditionnel basé sur l'argent mobile pour l'utilisation des soins de santé entre mars 2021 et juillet 2022. Les lignes pointillées bleues marquent la fin de l'intervention dans chaque établissement. Madagascar, 2021-2022*

L'établissement qui a enregistré la plus forte baisse du nombre de patients après la fin de l'intervention est l'établissement 10, un établissement privé situé dans une zone urbaine. De manière générale, les effets négatifs de la fin de l'intervention ont été les plus prononcés pour les soins de maternité, de pédiatrie et de chirurgie. Les consultations externes ont été moins affectées par la fin de l'intervention.

Le tableau 1 ci-dessous présente les résultats détaillés des modèles ITS. Les valeurs de changement d'échelon indiquent la différence immédiate dans le nombre de patients à la fin de l'intervention, tandis que le changement de pente décrit le changement dans le nombre de patients par mois au fil du temps.

*Tableau 1 : Résultats de l'analyse des séries temporelles interrompues à l'aide de la régression linéaire interrompue, évaluant les changements dans l'utilisation des soins de santé mesurés par le nombre de patients par mois dans onze établissements de santé du sud de Madagascar après la fin d'une intervention de transfert monétaire conditionnel basé sur l'argent mobile, mars 2021-juillet 2022 ; a = résultats statistiquement significatifs, définis par une valeur P < 0,05. Madagascar, 2021-2022.*

| **Établissement**  ***Catégories*** | **Variation du nombre de patients par mois à la fin de l'intervention (valeur P)** | **Changement de pente du nombre de patients par mois à la fin de l'intervention (valeur P)** |
| --- | --- | --- |
| **Établissement 1** |  |  |
| ***Tous les patients*** | **-2.84 (.02)^a^** | **-13.134 (<.01)^a^** |
| *Consultations externes* | -0.61 (.56) | -3.77 (<.01)^a^ |
| *Médecine interne* | -2.75 (.02)^a^ | -13.59 (<.01)^a^ |
| *Soins chirurgicaux* | -9.10 (<.01)^a^ | -53.94 (<.01)^a^ |
| *Maternité* | -3.08 (.01)^a^ | -16.75 (<.01)^a^ |
| *Soins pédiatriques* | -2.86 (.02)^a^ | -14.60 (<.01)^a^ |
|  |  |  |
| **Établissement 2** |  |  |
| ***Tous les patients*** | **0.22 (.83)** | **-9.43 (<.01)^a^** |
| *Consultations externes* | -4.83 (<.01)^a^ | -14.09 (<.01)^a^ |
| *Médecine interne* | 0.30 (.77) | -7.51 (<.01)^a^ |
| *Maternité* | -3.78 (<.01)^a^ | -14.38 (<.01)^a^ |
| *Soins pédiatriques* | 1.34 (.20) | -3.71 (<.01)^a^ |
|  |  |  |
| **Établissement 3** |  |  |
| ***Tous les patients*** | **-4.12 (<.01)^a^** | **-15.95 (<.01)^a^** |
| *Consultations externes* | -2.36 (.04)^a^ | -9.17 (<.01)^a^ |
| *Médecine interne* | -4.69 (<.01)^a^ | -7.26 (<.01)^a^ |
| *Soins chirurgicaux* | -3.66 (<.01)^a^ | -21.22 (<.01)^a^ |
| *Maternité* | -0.18 (.86) | -15.28 (<.01)^a^ |
| *Soins pédiatriques* | -1.82 (.10) | -7.17 (<.01)^a^ |
|  |  |  |
| **Établissement 4** |  |  |
| ***Tous les patients*** | **-1.77 (.09)** | **-1.35 (.19)** |
| *Consultations externes* | -1.77 (.09) | -1.35 (.19) |
| *Médecine interne* | -1.35 (.19) | -1.14 (.27) |
| *Soins pédiatriques* | -2.69 (.02)^a^ | -1.47 (.16) |
|  |  |  |
| **Établissement 5** |  |  |
| ***Tous les patients*** | **0.69 (.49)** | **-0.27 (.79)** |
| *Consultations externes* | 0.71 (.49) | -0.24 (.81) |
| *Maternité* | -0.65 (.53) | -1.79 (.09) |
| **Établissement 6** |  |  |
| ***Tous les patients*** | **-0.01 (.99)** | **1.24 (.23)** |
| **Établissement 7** |  |  |
| ***Tous les patients*** | **-2.12 (.06)** | **-11.88 (<.01)^a^** |
| *Consultations externes* | -1.34(.21) | -4.99 (<.01)^a^ |
| *Médecine interne* | -3.33 (<.01)^a^ | -14.55 (<.01)^a^ |
| *Soins chirurgicaux* | 2.42 (.04)^a^ | -6.74 (<01)^a^ |
| *Maternité* | -0.70 (.49) | -8.51 (<.01)^a^ |
| *Soins pédiatriques* | -2.49 (.03)^a^ | -20.23 (<.01)^a^ |
| **Établissement 8** |  |  |
| ***Tous les patients*** | **1.01 (.33)** | **-0.79 (.43)** |
| *Consultations externes* | 0.82 (.42) | -0.77 (.45) |
| *Médecine interne* | 0.21 (.83) | 1.97 (.06) |
| *Soins chirurgicaux* | -0.39 (.69) | -0.33 (.75) |
| *Maternité* | 0.99 (.33) | 0.93 (.37) |
| *Soins pédiatriques* | -3.65 (<.01) * | -3.07 (<.01)^a^ |
| **Établissement 9** |  |  |
| ***Tous les patients*** | **-1.12 (.29)** | **-1.38 (.19)** |
| *Outpatient consultations* | -0.41 (.69) | 0.19 (.86) |
| *Médecine interne* | -0.79 (.45) | -3.08 (.01)^a^ |
| *Soins chirurgicaux* | -1.72 (.12) | -1.64 (.13) |
| *Maternité* | -1.71 (.12) | 2.06 (.07) |
| *Soins pédiatriques* | -0.99 (.34) | 0.34 (.74) |
| **Établissement 10** |  |  |
| ***Tous les patients*** | **-7.13 (<.01)^a^** | **-49.69 (<.01)^a^** |
| *Consultations externes* | -2.03 (.07) | -12.65 (<.01)^a^ |
| *Médecine interne* | -3.67 (<.01)^a^ | -23.29 (<.01)^a^ |
| *Soins chirurgicaux* | -0.14 (.89) | -8.50 (<.01)^a^ |
| *Maternité* | -3.17 (<.01)^a^ | -28.98 (<.01)^a^ |
| *Soins pédiatriques* | -5.43 (<.01)^a^ | -21.03 (<.01)^a^ |
| **Établissement 11** |  |  |
| ***Tous les patients*** | **-1.90 (.07)** | **-2.72 (.01)^a^** |
| *Consultations externes* | -0.07 (.95) | -4.74 (<.01)^a^ |
| *Médecine interne* | -2.61 (.02)^a^ | -1.69 (.11) |
| *Soins chirurgicaux* | 0.91 (.38) | -2.14 (.04)^a^ |
| *Maternité* | -0.51 (.62) | -3.69 (<.01)^a^ |
| *Soins pédiatriques* | -0.88 (.39) | -2.62 (.02)^a^ |

**Résultats qualitatifs explicatifs.**

Les entretiens qualitatifs ont mis en évidence sept facteurs clés qui diffèrent entre les établissements où la fin de l'intervention a eu un impact significatif sur l'utilisation des soins de santé et ceux où cela n'a pas été le cas. Ces facteurs s'étendent sur trois niveaux : le niveau de la communauté, le niveau de l'établissement et le niveau de l'ONG chargée de la mise en œuvre. Le tableau 2 ci-dessous résume ces facteurs.

*Tableau 2 : Facteurs clés influençant la réussite de la mise en œuvre de l'intervention de transfert monétaire numérique, tels que déterminés dans l'analyse qualitative des entretiens approfondis avec les principaux acteurs de la mise en œuvre, classés par facteurs et par établissements avec et sans changements significatifs dans l'utilisation des soins de santé après la fin de l'intervention de transfert monétaire conditionnel, F = établissement. Madagascar, 2021-2022.*

| **FACTEURS CLÉS** | **INSTALLATIONS AYANT UN EFFET SIGNIFICATIF** (F1, F2, F3, F10, F11) | **INSTALLATIONS SANS EFFET SIGNIFICATIF** (F4, F5, F6, F8, F9) |
| --- | --- | --- |
| **Niveau communautaire** | | |
| Sensibilisation de la communauté | Lorsque la connaissance de l'intervention était élevée, les membres de la communauté étaient plus enclins à se faire soigner. | Un manque de connaissances sur l'intervention a entraîné de la confusion, de l'insécurité et une hésitation à recourir aux soins de santé. |
| **Niveau de l'établissement** | | |
| Motivation du personnel de l'établissement | Les établissements dont le personnel fait preuve d'une forte motivation intrinsèque et d'un état d'esprit altruiste obtiennent de meilleurs résultats et s'adaptent mieux aux défis programmatiques. | Les structures où le personnel exprime une motivation intrinsèque plus faible, s'adaptent moins bien aux défis programmatiques et obtiennent de moins bons résultats. |
| Primes versées par l'ONG | Les primes sont perçues comme utiles pour accroître la motivation | Les primes sont jugées insuffisantes et démotivantes, surtout lorsqu'elles sont inégalement réparties entre les membres du personnel de la structure. |
| Activités préexistantes de la même ONG chargée de la mise en œuvre | Les structures n'ayant pas bénéficié d'interventions antérieures de la part de la même ONG n'ont pas connu de confusion au sujet des interventions antérieures, ce qui n'a donc pas eu d'impact sur leur travail. | La confusion concernant les chevauchements et les différences entre l'intervention de transfert conditionnel d'argent et les activités antérieures de l'ONG a entraîné un manque de clarté pour les prestataires de soins de santé. |
| Culture numérique du personnel de la structure | Un niveau élevé de connaissances numériques parmi le personnel de la structure a eu un impact positif sur l'adoption de l'intervention. | Un faible niveau de culture numérique parmi le personnel de la structure a eu un impact négatif sur la participation à l'intervention. |
| **Niveau de l’ONG** | | |
| Personnel de l'ONG présent dans chaque établissement | La présence du personnel de l'ONG dans chaque établissement est perçue comme utile | Difficultés rencontrées en cas d'absence du personnel de l'ONG, par exemple pendant les week-ends ou lorsqu'il dessert plusieurs établissements |
| Rapidité et facilité des processus administratifs de l'intervention | Les établissements qui ont été intégrés plus tard dans l'intervention ont bénéficié d'une diminution des défis, car les problèmes fréquents rencontrés au cours des premiers mois de l'intervention ont été résolus. | Les retards et les difficultés liés au processus programmatique de l'intervention (en particulier la validation des demandes et le remboursement) ont constitué des obstacles majeurs, en particulier pour les premiers établissements intégrés. |

Niveau communautaire
*Sensibilisation de la communauté*

Pour les bénéficiaires, les différentes perceptions de la sensibilisation effectuée et leurs connaissances ultérieures sur l'intervention ont influencé l'utilisation des soins de santé. La sensibilisation à l'intervention a été principalement menée par les agents de santé communautaire, qui ont organisé des discussions de groupe, des activités de sensibilisation de masse (par exemple, les jours de marché) ou des visites à domicile et ont expliqué l'intervention et ses procédures à la population. Ces activités étaient généralement combinées à des activités d'éducation à la santé menées par les ASC. En outre, de brefs spots radio décrivant l'intervention ont été diffusés dans la zone d'intervention. Autour des établissements les plus performants, les patients ont déclaré que le fait d'avoir entendu parler de l'intervention par les ASC ou à la radio les avait encouragés à se faire soigner. À proximité des établissements moins performants, les participants ont fait part d'un manque d'informations sur l'intervention et n'en ont souvent entendu parler qu'une fois arrivés dans un établissement de santé.

*« C'est un agent de santé communautaire qui m'a parlé et m'a expliqué l'intervention (il a dit) que nous devons aller à l'hôpital quand nous sommes malades et ne pas aller n'importe où (ailleurs) pour recevoir des soins ». (Bénéficiaire 5)*

*« Il n'y a pas de sensibilisation ici (...). Je n'ai pas entendu parler (de l'intervention) ». (Non-bénéficiaire 7)*

Niveau de l'établissement
*Motivation du personnel de l'établissement*

La motivation intrinsèque des prestataires de soins différait d'un établissement à l'autre.Les établissements qui avaient activement cherché à participer à l'intervention et où les prestataires de soins avaient un grand intérêt à ce qu'elle réussisse ont mieux réussi dans l'intervention.Dans ces établissements, les prestataires de soins ont pris des mesures considérables pour assurer le fonctionnement de l'intervention, notamment en prenant en charge le ticket modérateur de leur poche ou en utilisant leur propre téléphone pour inscrire les patients.

*« Pour les établissements qui l'ont reçu involontairement, nous avons constaté plusieurs types de problèmes car, comme pour tout projet, s'il n'y a pas d'engagement de la part des personnes, il n'est pas possible de le mener à bien. Pour les établissements qui l'ont accepté : (...) ces établissements l'ont accueilli avec leur cœur, et nous avons immédiatement vu que le projet fonctionnait bien et qu'il apportait du bien à l'ensemble de l'établissement ».(Personnel chargé de la mise en œuvre du projet 6)*

*Primes versées par l'ONG*

Dans le cadre de l'intervention, les établissements ont reçu une prime pour chaque demande de remboursement. Dans certains établissements, les primes ont été perçues comme utiles pour accroître la motivation des prestataires de soins, tandis que d'autres ont estimé qu'elles étaient insuffisantes.

La façon dont les primes étaient distribuées aux membres du personnel (c'est-à-dire si chacun recevait une part ou si l'argent était conservé par la personne chargée de déposer les demandes) a également contribué à la frustration concernant les primes et à la baisse des performances dans certains établissements.

*« Les proches collaborateurs de (l'intervention) en ont profité parce qu'ils ont reçu des indemnités et qu'ils ont bien fait leur travail. (...). Je les ai vus courir partout pour faire leur travail et ils l'ont fait parfaitement. (prestataire de soins 3)*

*Au moment de (l'intervention), le travail était volumineux, l'indemnité pour les agents n'est pas suffisante. C'est le problème (...) Nous travaillons ici, nous travaillons la nuit quand il y a beaucoup de patients » (prestataire de soins 15)*

*Activités préexistantes de la même ONG chargée de la mise en œuvre*

De manière générale, les établissements où l'intervention a mieux réussi à augmenter l'utilisation des soins de santé sont ceux qui n'avaient pas collaboré auparavant avec la même ONG. Les établissements qui avaient déjà collaboré avec l'ONG ont exprimé une certaine confusion quant aux différences entre la nouvelle intervention et les interventions que l'ONG avait mises en œuvre précédemment.Les données ont en outre révélé une certaine réticence à s'adapter aux processus de la nouvelle intervention de la part de certains membres du personnel de l'établissement qui avaient travaillé sur ces projets antérieurs.

*Culture numérique du personnel de l'établissement*

Le degré de confiance et de compétence des prestataires de soins de santé et des employés administratifs vis-à-vis des compétences numériques nécessaires à l'administration de l'intervention a également influencé le succès de l'intervention.Les établissements dont le personnel avait un niveau élevé de culture numérique ont trouvé l'intervention plus facile à mettre en œuvre que ceux où les prestataires de soins de santé avaient un niveau de culture numérique plus faible.

Niveau des ONG

*Personnel de l'ONG présent dans chaque établissement*

Dans les établissements les plus performants, les membres actifs du personnel de l’ONG ont encouragé l'adoption de l'intervention par les patients et ont aidé les établissements à gérer la charge de travail associée à l'intervention.En raison de ce rôle positif du personnel de l’ONG, leur absence a été perçue comme problématique. Certains membres du personnel de l’ONG desservaient plusieurs établissements, ce qui a posé des problèmes aux établissements les jours où le personnel des ONG était absent. Les urgences survenant dans les centres pendant la nuit ou les week-ends ont été perçues comme tout aussi difficiles à gérer en raison de l'absence du personnel des ONG.

En outre, le personnel de l'ONG placé dans les établissements recevait un salaire de l'ONG, alors que les employés de l'établissement qui soutenaient également l'intervention ne recevaient pas de salaire supplémentaire, ce qui a suscité le mécontentement de certains prestataires de soins de santé.

*« J'encourage chaque patient qui arrive à discuter directement avec le médecin et à lui dire que nous avons entendu ceci et cela.(...) Je n'ai pas non plus adopté une attitude attentiste, mais j'ai tout fait, même si ce n'est pas mon travail, pour gagner le cœur de ces établissements, afin qu'ils reconnaissent que je suis enthousiaste à l'idée de collaborer ».*

*(Personnel chargé de la mise en œuvre du projet 7)*

*Rapidité et facilité des processus administratifs de l'intervention*

Les établissements où l'intervention a eu un impact significatif sur le nombre de patients étaient des établissements qui ont rejoint l'intervention plus tard, bénéficiant ainsi probablement de processus mieux établis au sein de l'ONG chargée de la mise en œuvre.

Les problèmes liés au remboursement des établissements par l'ONG ont eu un impact significatif sur les établissements, car les retards de paiement ont entraîné des problèmes pour les établissements liés au paiement des salaires de leur personnel ou à leur capacité à effectuer des prépaiements pour la commande de médicaments et de consommables. Il s'agit d'une source importante de frustration.

*« Le paiement effectué par (l'ONG) a été un peu retardé au début. C'était difficile pour l'hôpital à ce moment-là. L'hôpital a besoin de fonctionner pendant que l'argent est bloqué. Le problème a été réglé plus tard et tout est rentré dans l'ordre ». (prestataire de soins 11).*

L'un des principaux facteurs à l'origine du retard de paiement est la qualité insuffisante des demandes déposées par les établissements, comme l'ont souligné à la fois les prestataires de soins et le personnel de l’ONG. Les prestataires de soins de santé ont indiqué qu'une formation supplémentaire ou des directives sur le dépôt des demandes auraient pu améliorer cette situation.

Enfin, au début de l'intervention, des changements ont dû être apportés à la plateforme mTOMADY pour l'adapter à l'intervention. Ces changements ont été perçus comme une source de confusion et un défi pour les établissements. La fréquence des changements a été réduite pendant la durée de l'intervention, ce qui signifie que les établissements qui ont été intégrés à l'intervention plus tard étaient moins susceptibles de rencontrer de tels problèmes.

**DISCUSSION**

Notre étude visait à décrire l'impact d'une intervention de transfert monétaire conditionnel basé sur l'argent mobile sur l'utilisation des soins de santé dans un contexte humanitaire, ainsi qu'à identifier les facteurs clés qui ont influencé le succès de l'intervention. De manière générale, notre étude a montré une baisse significative de l'utilisation des soins de santé après la fin d'une intervention de transfert conditionnel d'argent mobile dans le sud de Madagascar. Les données provenant de onze établissements ont montré une réduction marquée des visites de patients, en particulier dans les services de maternité, de pédiatrie et de chirurgie, les consultations externes étant les moins touchées. Les résultats qualitatifs ont mis en évidence les facteurs clés influençant les résultats de l'intervention à trois niveaux. Ces facteurs comprennent la sensibilisation de la communauté, la motivation du personnel de l'établissement, la facilité des processus administratifs de l'intervention, la culture numérique du personnel de l'établissement et la présence du personnel de l'ONG dans l'établissement.

Notre analyse a montré que l'utilisation des soins de santé a diminué dans la plupart des établissements lorsque l'intervention de transfert conditionnel d'argent a pris fin. Cette constatation rejoint d'autres résultats obtenus à Madagascar, qui ont montré une augmentation de l'utilisation des soins de santé lorsque les frais d'utilisation ont été supprimés [24]. Ce changement a été le plus prononcé pour trois catégories de patients : les mères, les enfants et les patients nécessitant une intervention chirurgicale. Étant donné que les soins chirurgicaux sont particulièrement coûteux et susceptibles d'entraîner des dépenses de santé catastrophiques en Afrique subsaharienne [25-27], cette évolution n'est pas surprenante. Nos résultats indiquent que les patients pourraient renoncer à des soins chirurgicaux nécessaires en l'absence de méthodes de financement de leurs soins. Les données relatives aux coûts des soins pédiatriques sont moins complètes. Cependant, plusieurs études menées en Afrique subsaharienne suggèrent que les coûts associés aux soins pédiatriques pour l'asthme, la chirurgie pédiatrique et la pneumonie pourraient être catastrophiques pour les ménages [27-29]. Les preuves de l'impact de la couverture (partielle) des coûts ou de la réduction des frais pour les services de santé pédiatriques manquent cependant.

Les données sur des interventions similaires utilisant des transferts monétaire digital pour l'utilisation des soins de santé dans des contextes humanitaires sont très limitées.

Contrairement aux résultats précédents [30], notre étude a révélé une diminution significative de l'utilisation des soins de santé après la fin d'une intervention de transfert d'argent liquide. Cependant, notre étude analyse une intervention de transfert monétaire conditionnel, alors que les études précédentes ont examiné des transferts monétaires inconditionnels [30, 31], dans lesquels l'argent peut avoir été utilisé à d'autres fins.

Nos résultats s'alignent toutefois sur des données provenant de contextes non humanitaires, dans lesquels l'utilisation des soins de santé a augmenté en présence d'interventions ciblées de transfert d'argent liquide [32,33], en particulier pour les services de maternité [34]. Les interventions de transfert d'argent mobile dans des contextes non humanitaires, notamment en combinaison avec d'autres interventions telles que l'éducation à la santé ou les rappels par SMS, ont permis d'améliorer les comportements sexuels à risque [35] et sont perçues comme utiles pour améliorer l'observance et la prise en charge des patients atteints de tuberculose [36]. Il convient de noter que la plupart des interventions de transfert d'argent mobile ont utilisé de la monnaie physique, ce qui limite la comparabilité de nos résultats [30-34].

Malgré l'impact global de l'intervention de transfert conditionnel d'argent mobile sur l'utilisation des soins de santé, l'impact n'était pas significatif dans tous les établissements. Nous avons identifié sept facteurs clés susceptibles d'expliquer ces différences entre les établissements.

Au niveau de la communauté, les différences en matière de sensibilisation et donc de connaissances de la population sur l'intervention entre les communautés ont été déterminantes. Pour cette intervention, la sensibilisation n'a été menée que dans certaines communautés. Là où l'ONG a mis en œuvre la sensibilisation de la communauté, elle l'a fait par l'intermédiaire des relais communautaires et des campagnes radiophoniques. Les agents de santé communautaires sont essentiels pour améliorer les connaissances en matière de santé, l'accès aux soins et les résultats sanitaires dans les communautés qu'ils desservent [37-38]. Comme nous n'avons pas interrogé les agents de santé communautaires de cet échantillon, nous ne pouvons pas élucider davantage les raisons des différences entre les actions de proximité qu'ils ont menées. Cependant, étant donné les facteurs qui influencent les performances des agents de santé communautaires, notamment la formation, la supervision, la rémunération et la charge de travail [38, 39], il est logique que des facteurs similaires aient été décisifs dans notre contexte.

Au niveau de l'établissement, la motivation et l'état d'esprit de l'établissement sont apparus comme des facteurs déterminants de la réussite de l'intervention. La motivation des prestataires a été décrite comme un facteur clé dans la mise en œuvre et l'adoption de nouvelles pratiques [38, 39]. Il convient de noter que l'intervention décrite a pu avoir un impact sur la motivation des prestataires et donc renforcer positivement ou négativement les niveaux de motivation préexistants. Par exemple, l'intervention, qui a entraîné une augmentation du nombre de patients dans l'établissement, a pu accroître la charge de travail du prestataire, qui, en fonction de sa capacité de gestion perçue, a été identifiée comme un facteur déterminant de la motivation du prestataire [39]. De même, l'intervention, qui exigeait des prestataires de soins de santé qu'ils assument des tâches supplémentaires, a pu avoir un impact négatif sur la motivation des prestataires [39].

Les primes, que les établissements recevaient pour chaque demande déposée, étaient perçues très différemment selon les établissements. Dans certains établissements où tous les membres du personnel recevaient des avantages sous forme de primes, celles-ci étaient perçues comme des facteurs de motivation. Cette constatation est conforme aux données recueillies dans d'autres contextes, qui ont montré que les primes peuvent renforcer la motivation des prestataires et, éventuellement, la qualité des soins [40, 41]. Cet effet dépend toutefois de la manière dont la distribution des primes est perçue par les prestataires de soins de santé. Les primes perçues comme injustes ou inéquitables diminuent la motivation des prestataires [41, 42, 44].

Un autre facteur clé a été les interventions antérieures des ONG dans les mêmes structures, qui ont entraîné une confusion dans les structures avec les changements dans les procédures de la nouvelle intervention. Étant donné le pourcentage important de services publics fournis par des ONG en Afrique subsaharienne [45], il conviendrait d'examiner de plus près les effets négatifs des interventions antérieures des ONG sur les interventions futures.

Étant donné la nature numérique de l'intervention de transfert conditionnel d'argent, le manque de connaissances numériques au niveau de l'établissement a été un facteur clé qui a entravé l'intervention. Cette constatation rejoint les conclusions d'autres études menées en Afrique subsaharienne [46-51]. Face à la numérisation croissante de la prestation de soins de santé, les gouvernements, les organisations non gouvernementales et les organisations multilatérales devraient investir dans l'amélioration des compétences numériques des prestataires de soins de santé afin d'éliminer un obstacle majeur à la mise en œuvre d'interventions de santé numériques à l'avenir.

Du côté de l’ONG chargée de la mise en œuvre, deux facteurs se sont révélés décisifs. Tout d'abord, le personnel de l'ONG placé dans chaque établissement a été perçu comme un facteur de réussite important pour l'intervention. Le personnel de l'ONG a allégé la charge de travail des prestataires de soins de santé en soutenant l'administration de l'intervention [52]. Malgré cet effet positif, le fait que le personnel de l'ONG soit payé directement par l'ONG peut avoir renforcé les inégalités perçues en matière de rémunération et donc accentué les frustrations préexistantes [44].

Un autre résultat notable de notre étude est que les établissements qui semblent avoir connu le plus de succès sont ceux où l'intervention a été mise en œuvre plus tard, et qui ont donc bénéficié de processus mieux établis et mieux rodés. Cela fait écho à des résultats antérieurs concernant des interventions de santé publique, qui ont identifié « l'infrastructure de mise en œuvre », y compris des systèmes administratifs robustes, comme un facteur clé de succès [40, 41]. Il est essentiel de prendre en compte ces défis pour les futures interventions similaires, par exemple en incorporant des phases de pilotage dans les plans de mise en œuvre afin de mettre en place des processus administratifs solides dès le début.

L'un des principaux défis des interventions de transferts monétaires, en particulier dans les situations humanitaires, est de maintenir l'augmentation de l'utilisation des soins de santé après la fin de l'aide financière. Sur la base des résultats de notre étude, nous proposons plusieurs stratégies pour améliorer la durabilité d'interventions similaires.

Tout d'abord, le renforcement des systèmes locaux de financement de la santé est essentiel pour maintenir l'utilisation des services. Les interventions futures pourraient s'associer aux gouvernements locaux, aux régimes d'assurance maladie privés ou à but non lucratif pour transformer les transferts monétaires conditionnels en une aide financière plus permanente pour les populations vulnérables. Le développement de mécanismes de subventions partielles par le biais de fonds de santé nationaux ou de régimes d'assurance maladie communautaires peut favoriser l'accès continu aux services essentiels sans dépendre uniquement d'un financement externe.

Deuxièmement, nous avons observé que l'augmentation de l'utilisation des soins de santé au cours de la période d'intervention était en partie due à des efforts ciblés de sensibilisation et d'éducation à la santé au sein des communautés. Le maintien des agents de la santé communautaires dans la promotion de la santé peut maintenir la sensibilisation de la communauté et la valeur perçue des services de santé. Les interventions futures devraient investir dans la formation des agents de la santé communautaires et créer des ressources pour permettre aux communautés de prendre des décisions éclairées en matière de santé, encourageant ainsi l'utilisation des soins de santé, même sans incitations financières directes.

Enfin, les données analysées dans le cadre de cette étude contenaient plusieurs indications selon lesquelles l'intervention avait un impact positif sur la confiance que les patients exprimaient à l'égard des prestataires de soins et du système de santé. C'est notamment le cas dans les établissements où les prestataires de soins ont aidé les patients à s'inscrire à l'intervention, par exemple en leur permettant d'utiliser leur carte d'identité pour obtenir une carte SIM, ou lorsqu'ils ont aidé les patients qui avaient du mal à payer leur ticket modérateur. Cette amélioration de la confiance est cruciale car elle peut conduire à des améliorations durables dans le comportement de recherche de soins de santé, même après les incitations financières. Les futurs responsables de la mise en œuvre devraient donner la priorité aux mesures de renforcement de la confiance, telles qu'une communication transparente, une sensibilisation continue et des soins centrés sur le patient, afin de cultiver un environnement de soins de santé dans lequel les patients se sentent soutenus et motivés pour rechercher une assistance médicale en temps opportun.

**Limites**

Nous avons réalisé notre série temporelle interrompue sans groupe de contrôle. Compte tenu des urgences de santé publique survenues dans le sud de Madagascar ces dernières années, qui ont eu un impact sur l'utilisation des soins de santé dans la région, nous avons opté pour un groupe de contrôle historique. En 2020, la pandémie de COVID-19 a fortement touché la population et le système de santé malgaches [7]. En 2018/2019, une grave épidémie de rougeole [49] et en 2017 une épidémie de peste ont touché l'île [52].

Le choix des données de contrôle provenant d'établissements de santé situés dans une autre partie du pays s'est avéré tout aussi peu fiable. Même si le système de santé est faible dans l'ensemble de Madagascar, le sud de l'île présente des défis particuliers. Les barrières géographiques d'accès sont particulièrement prononcées [8], et les installations, les ressources humaines et les équipements sont particulièrement rares [10]. De même, l'extrême pauvreté est plus prononcée dans les régions étudiées que dans le reste du pays [8, 55]. Enfin, la grande sécheresse et la famine qui s'en est suivie ont été spécifiques à la région étudiée et n'ont pas touché les autres parties du pays [9].

Quatre établissements de santé sur quinze ont refusé de partager leurs registres avec nous pour l'analyse quantitative. Comme les établissements n'étaient pas tenus de justifier leur refus de partager leurs données, nous ne pouvons pas éliminer la possibilité d'un biais de sélection dans notre échantillon final. Cependant, nous avons inclus des établissements dont l'impact de l'intervention sur l'utilisation des soins de santé était variable, ce qui suggère que les résultats globaux ne sont pas faussés.

Nos données qualitatives proviennent d'un échantillon de commodité d'établissements, qui comprend la plupart des établissements pour lesquels une analyse quantitative a été menée, mais pas tous, ce qui signifie que nous n'avons peut-être pas saisi tous les facteurs qui ont influencé le succès de l'intervention.

De plus, nos données qualitatives ont été collectées en malgache et traduites en anglais, ce qui peut avoir introduit des erreurs de traduction. Cependant, toutes les données ont été traduites par un interprète formé ayant une expérience préalable de la recherche qualitative et vérifiées au hasard par un locuteur natif malgache pour s'assurer de la cohérence entre l'enregistrement et la traduction.

**Conclusions de l'étude**

En conclusion, notre étude montre qu'une intervention de transfert monétaire conditionnel basé sur l'argent mobile a conduit à une augmentation de l'utilisation des soins de santé dans un contexte humanitaire, en particulier pour les cas chirurgicaux et pédiatriques. Cela indique que des interventions similaires pourraient être utiles pour atténuer les effets de futures crises humanitaires sur l'utilisation des soins de santé.

Plusieurs facteurs ont influencé le succès de l'intervention. Les concepteurs et les responsables de la mise en œuvre de futures interventions similaires devraient atténuer ces facteurs de manière proactive. D'autres chercheurs devraient s'efforcer de combler les lacunes qui subsistent en ce qui concerne l'utilisation d'interventions de transfert monétaire basées sur l'utilisation de l'argent mobile dans les situations humanitaires.

**REMERCIEMENTS**

Nous tenons à remercier toutes les personnes qui ont participé à nos entretiens et qui ont partagé leurs points de vue, leurs idées et leurs pensées ouvertement et honnêtement. Nous tenons également à remercier toute l'équipe de Médecins pour Madagascar pour son soutien à notre recherche et pour avoir rendu cette intervention possible. Nous tenons également à remercier le chercheur local qui a mené la collecte des données qualitatives pour son dévouement et pour avoir rendu cette étude possible.

**CONFLITS D'INTÉRÊTS**

Les auteurs ne déclarent aucun conflit d'intérêt

**DÉCLARATION DE FINANCEMENT**

La Gesellschaft für Internationale Zusammenarbeit [Coopération Allemande pour le Développement International] (GIZ) a sponsorisé l'intervention analysée dans cette étude. Une subvention de la Fondation Theracur a spécifiquement soutenu les activités de recherche.

Multimédia Annexe 1 :

Localisation des établissements de santé dans le sud de Madagascar qui ont participé à une intervention de transfert monétaire conditionnel basé sur l'argent mobile pour l'utilisation des soins de santé entre mars 2021 et juillet 2022. F= Établissement.

Les établissements situés dans les zones urbaines offrant des soins secondaires sont marqués par des points bleus, les établissements situés dans les zones rurales offrant des soins secondaires sont marqués par des triangles rouges, et les établissements situés dans les zones urbaines offrant des soins primaires sont marqués par des carrés verts. La capitale, Antananarivo, est indiquée en noir. (Plusieurs structures (F4, F5, F10) se trouvaient dans la ville de Tolagnaro, Fort-Dauphin, dans le sud-est de Madagascar, ce qui a conduit à un regroupement des symboles dans la région). Madagascar, 2021-2022.

Annexe multimédia 2 :

Niveau de soins, cadre, propriété et période d'intervention de onze des quinze établissements de santé du sud de Madagascar qui ont participé à une intervention de transfert monétaire conditionnel basé sur l'argent mobile pour l'utilisation des soins de santé entre mars 2021 et juillet 2022. Les quatre établissements restants ont refusé de partager les données pour l'analyse et ont été exclus de cette étude. Madagascar, 2021-2022.

Annexe multimédia 3: Traduction française du manuscrit

RÉFÉRENCES

1. International Monetary Fund (IMF). Digital Currency Innovations in Sub-Saharan Africa. In: Regional Economic Outlook: Sub-Saharan Africa—Living on the Edge. Washington, DC; October 2022.

2. GSMA. State of the Industry Report on Mobile Money 2023. Available online at: https://www.gsma.com/mobilefordevelopment/resources/state-of-the-industry-report-on-mobile-money-2023-2/. Accessed February 16, 2024.

3. Garcia M. The Cash Dividend: The Rise of Cash Transfer Programs in Sub-Saharan Africa. Washington, DC: World Bank; 2022.

4. Joint United Nations Programme on HIV/AIDS. Mitigating the Socioeconomic Consequences of COVID-19 in Burkina Faso: Rapid Cash Transfers for Vulnerable People Living with HIV and Key Populations. Geneva; 2022. Licence: CC BY-NC-SA 3.0 IGO.

5. Ahmed H, Cowan B. Mobile money, and healthcare use: Evidence from East Africa. World Development. 2021;141:105392.

6. The World Bank. Madagascar. Available online at: https://data.worldbank.org/country/madagascar. Accessed January 2, 2024.

7. Harisoa R, David BJ. The economic impact of COVID-19 on African countries: The case of Madagascar. SHS Web Conf. 2023;163:01039. doi:10.1051/shsconf/202316301039

8. Institut National de la Statistique. Troisième Recensement Général de la Population et de l’Habitation. Antananarivo, Madagascar; 2019.

9. World Food Programme. 2022 Annual Country Report Highlights. Available online at: https://docs.wfp.org/api/documents/WFP-0000148580/download/. Accessed January 2, 2024.

10. Ministère de la Santé Publique. Plan de Développement du Secteur Santé 2020-2024. Antananarivo, Madagascar; 2019.

11. Ataguba JE. COVID-19 pandemic, a war to be won: Understanding its economic implications for Africa. Appl Health Econ Health Policy. 2020;18(3):325–328. PMID: 32249362.

12. Kairu A, Orangi S, Mbuthia B, et al. The impact of COVID-19 on health financing in Kenya. PLOS Glob Public Health. 2023;3(10):e0001852. PMID: 37889878.

13. United Nations. Human Development Index. Available online at: https://hdr.undp.org/data-center/human-development-index. Accessed January 2, 2024.

14. Lang E, Saint-Firmin P, Olivetti A, et al. Analyse du Système de Financement de la Santé à Madagascar pour Guider de Futures Réformes, Notamment la CSU. Washington, DC: Palladium, Health Policy Plus; 2018.

15. World Health Organization. Madagascar. Available online at: https://www.who.int/countries/mdg. Accessed January 2, 2024.

16. mTOMADY. Powering Access to Healthcare. Available online at: https://www.mtomady.com/. Accessed May 13, 2024.

17. Creswell JW, Plano Clark VL, Westfall GJ. Designing and Conducting Mixed Methods Research. 3rd ed. Sage Publications; 2017.

18. Ali MM. Durbin-Watson and generalized Durbin-Watson tests for autocorrelations and randomness. J Bus Econ Stat.1987;5(2):195–203.

19. RStudio Team. RStudio: Integrated Development for R.* Boston, MA: RStudio, PBC; 2020. Available online at:<http://www.rstudio.com/>. Accessed February 27, 2024.

20. Braun V, Clarke V. Using thematic analysis in psychology. Qual Res Psychol. 2006;3(2):77-101. doi:10.1191/1478088706qp063oa

21. Dhakal K. NVivo. J Med Libr Assoc. 2022;110(2):270-272. doi:10.5195/jmla.2022.1271

22. von Elm E, Altman DG, Egger M, Pocock SJ, Gøtzsche PC, Vandenbroucke JP; STROBE Initiative. The Strengthening the Reporting of Observational Studies in Epidemiology (STROBE)statement: guidelines for reporting observational studies. J Clin Epidemiol. 2008 Apr;61(4):344-9. PMID: 18313558

23. O'Brien BC, Harris IB, Beckman TJ, Reed DA, Cook DA. Standards for reporting qualitative research: a synthesis of recommendations. Acad Med. 2014;89(9):1245-1251.

24. Garchitorena A, Miller AC, Cordier LF, et al. In Madagascar, use of healthcare services increased when fees were removed: Lessons for universal health coverage. Health Aff (Millwood). 2017;36(8):1443–1451. PMID: 28784737.

25. Shrime MG, Dare A, Alkire BC, et al. A global country-level comparison of the financial burden of surgery. Br J Surg. 2016;103(11):1453–1461. PMID: 27428044.

26. Okoroh JS, Riviello R. Challenges in healthcare financing for surgery in sub-Saharan Africa. Pan Afr Med J. 2021;38:198. doi:10.11604/pamj.2021.38.198.27115. PMID: 33995804.

27. Yap A, Olatunji BT, Negash S, et al. Out-of-pocket costs and catastrophic healthcare expenditure for families of children requiring surgery in sub-Saharan Africa. Surgery. 2023;174(3):567–573. PMID: 37385869.

28. Zhang S, Sammon PM, King I, et al. Cost of management of severe pneumonia in young children: systematic analysis. J Glob Health. 2016;6(1):010408. PMID: 27231544.

29. Ughasoro MD, Eze JN, Ayuk AC, et al. Economic burden of childhood asthma in children attending a follow-up clinic in a resource-poor setting of Southeast Nigeria. Paediatr Respir Rev. 2021;37:74–79. PMID: 32169438.

30. Pega F, Liu SY, Walter S, Lhachimi SK. Unconditional cash transfers for assistance in humanitarian disasters: Effect on use of health services and health outcomes in low- and middle-income countries. Cochrane Database Syst Rev. 2015;(9):CD011247. doi:10.1002/14651858.CD011247.pub2. PMID: 26360970.

31. Tappis H, Doocy S. The effectiveness and value for money of cash-based humanitarian assistance: a systematic review. J Dev Eff. 2018;10(1):121–144.

32. Mesmar S, Talhouk R, Akik C, et al. The impact of digital technology on health of populations affected by humanitarian crises: Recent innovations and current gaps. J Public Health Policy. 2016;37(2):167–200. PMID: 27899794.

33. Briaux J, Martin-Prevel Y, Carles S, et al. Evaluation of an unconditional cash transfer program targeting children’s first 1,000 days’ linear growth in rural Togo: A cluster-randomized controlled trial. PLOS Med. 2020;17(11):e1003388. PMID: 33201927.

34. Hunter BM, Harrison S, Portela A, Bick D. The effects of cash transfers and vouchers on the use and quality of maternity care services: A systematic review. PLOS One. 2017;12(3):e0173068. PMID: 28328940.

35. Kuringe E, Christensen A, Materu J, Drake M, Majani E, Casalini C, Mjungu D, Mbita G, Kalage E, Komba A, Nyato D, Nnko S, Shao A, Changalucha J, Wambura M. Effectiveness of Cash Transfer Delivered Along With Combination HIV Prevention Interventions in Reducing the Risky Sexual Behavior of Adolescent Girls and Young Women in Tanzania: Cluster Randomized Controlled Trial. JMIR Public Health Surveill 2022;8(9):e30372. doi: 10.2196/30372. PMID: 36121686.

36. Musiimenta A, Tumuhimbise W, Atukunda E, Mugaba A, Linnemayr S, Haberer J. Digital Adherence Technologies and Mobile Money Incentives for Management of Tuberculosis Medication Among People Living With Tuberculosis: Mixed Methods Formative Study. JMIR Form Res 2023;7:e45301. doi: 10.2196/45301. PMID: 37043263.

37. World Health Organization. What Do We Know About Community Health Workers? A Systematic Review of Existing Reviews. Geneva; 2020 (Human Resources for Health Observer Series No. 19). Licence: CC BY-NC-SA 3.0 IGO. Available online at: https://creativecommons.org/licenses/by-nc-sa/3.0/igo.

38. Swider SM. Outcome effectiveness of community health workers: An integrative literature review. Public Health Nurs. 2002;19(1):11–20.PMID: 11841678.

39. Scott K, Beckham SW, Gross M, et al. What do we know about community-based health worker programs? A systematic review of existing reviews on community health workers. Hum Resour Health. 2018;16(1):39. PMID: 30115074.

40. Milat AJ, Bauman A, Redman S. Narrative review of models and success factors for scaling up public health interventions. Implement Sci. 2015;10(1):113. PMID: 26264351.

41. Bulthuis SE, Kok MC, Raven J, et al. Factors influencing the scale-up of public health interventions in low- and middle-income countries: A qualitative systematic literature review. Health Policy Plan. 2020;35(2):219–234. PMID: 31722382.

42. Ergo A, Paina L, Morgan L, et al. Creating stronger incentives for high-quality healthcare in low- and middle-income countries. Washington, D.C., March 2012

43. Okonofua F, Ntoimo LF, Yaya S, et al. Effect of a multifaceted intervention on the utilisation of primary health for maternal and child healthcare in rural Nigeria: A quasi-experimental study. BMJ Open. 2022;12(2):e049499. PMID: 35135763.

44. Chimhutu V, Songstad NG, Tjomsland J, et al. The inescapable question of fairness in pay-for-performance bonus distribution: A qualitative study of health workers’ experiences in Tanzania. Glob Health. 2016;12(1):77. PMID: 27884185.

45. Nega B, Schneider G. NGOs, the state, and development in Africa. Rev Soc Econ. 2014;72(4):485–503.

46. Granja C, Janssen W, Johansen MA. Factors determining the success and failure of eHealth interventions: Systematic review of the literature. J Med Internet Res.2018;20(5):e10235. doi:10.2196/10235. PMID: 29716883.

47. Okano JT, Ponce J, Krönke M, et al. Lack of ownership of mobile phones could hinder the rollout of mHealth interventions in Africa. eLife. 2022;11:e79615. PMID: 36255055.

48. Kanjo C, Hara J, Kaasbøll J. Digital empowerment for health workers, and implications on EMRs utilisation. J Health Inform Afr. 2019;6(2):74–83.

49. Chereka AA, Demsash AW, Ngusie HS, et al. Digital health literacy to share COVID-19 related information and associated factors among healthcare providers worked at COVID-19 treatment centres in Amhara region, Ethiopia: A cross-sectional survey. Informatics Med Unlocked.*2022;30:100934. PMID: 35441087.

50. Tegegne MD, Tilahun B, Mamuye A, et al. Digital literacy level and associated factors among health professionals in a referral and teaching hospital: An implication for future digital health systems implementation. Front Public Health. 2023;11:1130894. doi:10.3389/fpubh.2023.1130894. PMID: 37113180.

51. Owoyemi A, Osuchukwu JI, Azubuike C, et al. Digital solutions for community and primary health workers: Lessons from implementations in Africa. Front Digit Health. 2022;4:876957. doi:10.3389/fdgth.2022.876957. PMID: 35754461.

52. Muthuri RNDK, Senkubuge F, Hongoro C. Determinants of motivation among healthcare workers in the East African Community between 2009–2019: A systematic review. Healthcare. 2020;8(2):164. PMID: 32532016.

53. Nimpa MM, Andrianirinarison JC, Sodjinou VD, et al. Measles outbreak in 2018-2019, Madagascar: Epidemiology and public health implications. Pan Afr Med J. 2020;35:84. PMID: 32537087.

54. World Health Organization. Plague – Madagascar (online). 2017. https://www.who.int/emergencies/disease-outbreak-news/item/15-november-2017-plague-madagascar-en. Accessed January 2, 2024.

55. Institut National de la Statistique et ICF. Enquête Démographique et de Santé à Madagascar, 2021. Antananarivo, Madagascar and Rockville, MD: INSTAT and ICF; 2022.
